# A bioinformatics pipeline for the design of a SART3-targeted cancer vaccine with enhanced immunogenicity

**DOI:** 10.1186/s44342-026-00068-5

**Published:** 2026-05-01

**Authors:** Zeynab Bayat, Faezeh Mahdian-Khoo, Lida Samie, Amir Taherkhani

**Affiliations:** 1 Department of Oral and Maxillofacial Medicine, Faculty of Dentistry, Hamadan University of Medical Sciences, Hamadan, Iran; 2 Urology and Nephrology Research Center, Avicenna Institute of Clinical Sciences, Hamadan University of Medical Sciences, Hamadan, Iran; 3 Research Center for Molecular Medicine, Institute of Cancer, Hamadan University of Medical Sciences, Hamadan, Iran

**Keywords:** Cancer, Epitopes, Immunoinformatics, SART3, T Lymphocyte, Vaccine

## Abstract

**Background and objectives:**

Squamous cell carcinoma antigen recognized by T-cells 3 (SART3) has emerged as a promising target for cancer immunotherapy, given its overexpression in various malignancies and low or absent expression in non-tumorous tissues. This study aimed to design rationally and in silico evaluate a multi-epitope T cell vaccine targeting SART3, incorporating a TLR4 agonist adjuvant. The vaccine’s predicted immunogenicity, physicochemical properties, structural stability, and interaction with TLR4 were comprehensively assessed. Additional assessments of cytokine-inducing potential, B-cell epitopes, and disulfide engineering opportunities were also executed.

**Methods:**

Potential T-cell epitopes from SART3 were identified using IEDB and screened for antigenicity (VaxiJen), toxicity (ToxinPred), and MHC-I/II binding affinity. Cytokine-inducing epitopes were evaluated using IL4pred, IL-10Pred, and IFNepitope servers. B-cell epitopes were predicted using ElliPro. The vaccine underwent comprehensive physicochemical, structural (I-TASSER/GalaxyRefine), molecular docking (HDOCK), molecular dynamics simulations, and disulfide engineering (Disulfide by Design 2.0) analyses.

**Results:**

The optimized 344-residue vaccine demonstrated non-allergenicity, high stability (instability index 17.16), antigenicity (Vaxijen 0.67), and solubility (SOLpro 0.96). HDOCK predicted favorable vaccine–TLR4 binding (ΔG = − 265.61 kcal/mol, confidence 91%). MD simulations confirmed complex stability. Cytokine analysis revealed the potential to induce IL-4 and IL-10. The Val80–Ala123 pair exhibited the lowest bond energy (1.16 kcal/mol), indicating the optimal geometry for disulfide bond formation. The in silico immune simulations demonstrated a robust immune response following vaccine administration.

**Conclusion:**

This rationally designed SART3-targeted multi-epitope vaccine exhibits promising in silico characteristics across immunogenicity, physicochemical, cytokine-inducing, B-cell epitope, structural, and disulfide engineering profiles, warranting experimental validation for cancer immunotherapy development.

**Supplementary Information:**

The online version contains supplementary material available at 10.1186/s44342-026-00068-5.

## Introduction

Squamous cell carcinoma antigen recognized by T-cells 3 (SART3) is a multifaceted nuclear protein with a complex structure. It binds to RNA and possesses diverse functionalities within the cell. Its intricate architecture features a half-tetracopeptide repeat (HAT) in the N-terminal region and two RNA recognition motifs (RRM1 and RRM2) near the C-terminus [1, 2]. SART3 plays a critical role in pre-mRNA splicing, a process essential for gene expression. It functions as a U4/U6 recycling factor, facilitating the efficient reuse of these components within the splicing machinery [3]. Beyond its role in splicing, SART3 influences a broader array of biological processes, including gene regulation [4–6], cancer immunology [7–9], maintenance of stem cell pluripotency [10, 11], embryonic development [12], and hematopoiesis [13, 14]. SART3 has emerged as a significant player in cancer. Its regulatory roles in miRNA biogenesis, immune checkpoint modulation, altered RNA splicing patterns, and hypoxia response mechanisms highlight its importance in tumorigenesis. SART3 overexpression is linked with a poorer prognosis in various cancers, such as hepatocellular carcinoma and non-small-cell lung cancer [15–19]. In addition, SART3 has been demonstrated as a tumor-associated antigen in multiple cancers, including breast cancer [20], hepatocellular carcinoma [21], renal cell carcinoma [22], brain tumors [23], prostate cancer [24], musculoskeletal tumors [25], and gastric cancers [26]. Although its expression is minimal in most normal tissues, it is consistently upregulated across a diverse range of solid tumors, including those of the breast, brain, kidney, and musculoskeletal system. This tumor-specific expression pattern has supported its development as a tumor-associated antigen for peptide-based immunotherapy [20, 22, 23, 25].

Multi-epitope vaccines represent a significant leap forward in cancer immunotherapy by addressing the inherent heterogeneity and complexity of tumor antigens. This innovative platform seeks to elicit a robust immune response by incorporating diverse antigenic epitopes. This multi-pronged approach broadens and deepens the immune response against cancer cells, leading to a potentially more effective attack, including epitopes targeting CD4 + helper T-cells and CD8 + cytotoxic T-cells, which appear critical for sustaining an antitumor immune response. CD4 + helper T-cells play a crucial role in orchestrating the immune response, while CD8 + cytotoxic T-cells directly eliminate cancer cells. Furthermore, integrating computational tools for epitope selection and exploring combinatorial approaches with checkpoint inhibitors or other immunomodulators holds immense promise. These advancements could maximize therapeutic outcomes across a broad spectrum of cancer types [27–30].

In recent years, the immunoinformatics-driven design of multi-epitope vaccines has emerged as a powerful alternative to purely empirical antigen discovery. By enabling rapid, high-throughput screening of large proteomes, these computational pipelines identify antigenic, non-allergenic, and non-toxic epitopes while substantially reducing the experimental workload and cost inherent in traditional trial-and-error approaches. The integration of multiple prediction tools further enriches the search for epitopes with favorable binding, safety, and physicochemical profiles, thereby increasing the likelihood that the final vaccine constructs will prove immunogenic and suitable for manufacturing upon laboratory validation. Notably, multiple vaccines developed through immunoinformatics approaches have been experimentally validated and have transitioned into effective clinical applications [31–34].

**Fig. 1:**
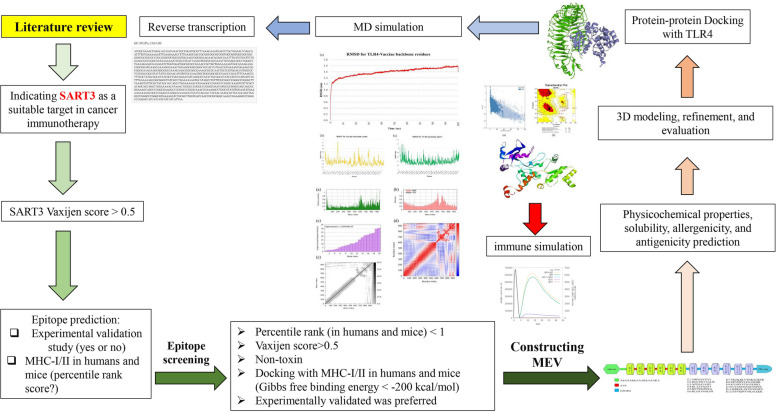
Schematic overview of the in silico workflow for designing a SART3-targeted multi-epitope vaccine. SART3, Squamous Cell Carcinoma Antigen Recognized by T-cells 3

The graphical abstract provides an overview of the in silico workflow employed to identify SART3-derived epitopes, design the multi-epitope vaccine construct, and assess its immunogenic properties (Fig. 1). Given the overexpression of SART3 across multiple malignancies and its potential as a tumor-associated antigen, a rationally designed multi-epitope vaccine targeting SART3-derived T-cell epitopes may offer a promising strategy for cancer immunotherapy. Accordingly, the primary objective of this study was to design an SART3-targeted multi-epitope vaccine using immunoinformatics tools to identify and prioritize high-affinity MHC class I and II epitopes, and to assemble them with an appropriate TLR4 agonist adjuvant and suitable linkers. A secondary objective was to comprehensively evaluate the resulting vaccine construct in silico, including its antigenicity, allergenicity, physicochemical properties, three-dimensional structure, predicted interaction with TLR4, immune simulation profile, and suitability for expression in *E. coli*.

## Materials and methods

### SART3 and adjuvant sequence acquisition

The vaccine construct design incorporates the amino acid sequence of the SART3 antigen, comprising 963 residues, retrieved from UniProtKB (Supplementary data 1) [35]. Notably, the 50S ribosomal protein L7/L12 was derived from Mycobacterium *tuberculosis* (*M.* *tuberculosis*) and was also integrated. Several studies have highlighted the strong binding affinity of this protein for Toll-like receptor 4 (TLR4), an essential pattern recognition receptor that triggers innate immune responses upon recognition of pathogen-associated molecular patterns. The inclusion of the 50S ribosomal protein L7/L12 from *M. tuberculosis* has been supported by research demonstrating its agonist activity with TLR4, thereby initiating the innate immune response [36, 37].

#### Major histocompatibility complex (MHC) class I and II epitopes

The Immune Epitope Database (IEDB) platform (https://tools.iedb.org/mhci/) [38] was employed to identify potential MHC class I and II epitopes within SART3. This computational tool evaluates candidate epitopes and ranks them by their binding strength to common human and mouse MHC class I and II alleles. While our research primarily focused on human applications, we also conducted a computational assessment of epitope binding to murine MHC-I and MHC-II molecules. This comprehensive approach aimed to identify epitopes with robust binding affinity in human and mouse models. This crucial aspect could influence the vaccine’s effectiveness across species and enhance our overall contribution to vaccine development research.

The IEDB repository was searched to augment the computational predictions for empirically confirmed MHC-I and MHC-II epitopes linked to SART3 (Supplementary data 2). The protocol for designing a multi-epitope vaccine incorporated a carefully selected blend of these validated epitopes, along with the predicted candidates. Notably, the most promising experimentally verified epitopes were evaluated extensively using diverse algorithms, elaborated upon in the subsequent section.

### Antigenic epitope screening

To determine the most immunogenic SART3 epitopes for MHC-I and MHC-II presentation, a comprehensive screening methodology was employed:

1. Initial filtering: epitopes with a percentile rank above 1.0 for human MHC alleles were eliminated, indicating weak predicted binding affinity. A percentile rank of < 1.0 was selected as a stringent cutoff to enrich for the top 1% of predicted MHC binders, consistent with IEDB recommendations for epitope selection and with prior multi-epitope vaccine design protocols employing the same threshold [39–42].

2. Antigenicity and toxicity evaluation: the filtered epitopes were analyzed using the Vaxijen v2.0 platform [43] to assess their immune response potential. The ToxinPred tool [44] was utilized to identify and exclude potential toxicity-associated epitopes.

3. Prioritization of promising candidates: epitopes that were experimentally validated or had a percentile rank below 1.0 for murine MHC alleles were given precedence, suggesting enhanced immunogenic potential.

### Final epitope selection approaches

#### Prediction-driven method

Epitopes with a murine MHC percentile rank under 1.0 underwent molecular docking analysis via the HPEPDOCK 2.0 platform [45]. This process evaluated their binding affinity to various MHC alleles, including human (HLA-A0201, HLA-B3501) and murine (H2-Kd, H2-Ld, H2-Dd). Epitopes exhibiting vital binding energies (< − 200 kcal/mol) were retained and further assessed based on their Vaxijen antigenicity score for final selection.

It should be noted that the docking binding energy threshold (≤ − 200 kcal/mol) employed in this study was selected based on the authors’ comparative and empirical experience rather than as a universally established cutoff. In the present work, this value served as a pragmatic filter to prioritize high-affinity complexes for downstream analysis. Moreover, this threshold has been consistently applied in our previous immunoinformatics studies, ensuring methodological continuity and facilitating internal comparison across investigations [41, 42].

### Validation-focused approach

A selection criterion similar to those used in method (a) was applied to identify top candidates for experimentally confirmed epitopes. However, given their established immunogenic properties, a murine MHC percentile rank below 1.0 for these epitopes was not mandated.

### Constructing a multi-epitope vaccine construct

Following the multi-step screening process, epitopes demonstrating superior rankings were selected as potential candidates for incorporation into the vaccine design. This selection focused on epitopes that can elicit potent, targeted immune responses.

The selected epitopes were subsequently assembled into unified constructs using appropriate linker sequences. The choice of these linkers was guided by information from specialized databases on multi-epitope peptide vaccines and pertinent scientific literature [46]. These linker sequences were recognized as crucial for enabling proper epitope presentation by MHC molecules and ensuring optimal spatial configurations within the final vaccine construct.

The N-terminal adjuvant was connected to the initial cytotoxic T lymphocyte (CTL) epitope using the AEAAAKEAAAKEAAAKA linker sequence, which is known to promote an alpha-helical secondary structure. This (EAAAK)₃ linker ensures structural stability and maintains a defined spatial orientation between the adjuvant and the epitope components [47]. The MHC-I epitopes were subsequently joined using the AAY linker. The final CTL epitope was then linked to the first helper T lymphocyte (HTL) epitope via the GPGPG linker [48]. The inclusion of AAY linkers between CTL epitopes has been previously reported to improve the antigenicity and conformational stability of multi-epitope vaccine constructs [49]. Similarly, the GPGPG linker has been shown to enhance immunogenic potential, flexibility, protein folding, and solubility [50]. This GPGPG linker was also employed for two additional purposes: joining the MHC-II epitopes and attaching the final HTL epitope to the C-terminal His-tag sequence [42].

A diverse array of 111 vaccine constructs with distinct primary sequences was randomly generated to evaluate the vaccine candidates comprehensively. This collection of vaccine designs was then subjected to further rigorous evaluation methodologies.

### Evaluating allergenicity and antigenicity responses of multi-epitope vaccines

The potential allergenicity of the vaccine candidates was evaluated using the online AllerTOP tool [51]. This web-based platform can be accessed at http://www.ddg-pharmfac.net/allertop/. Additionally, the antigenicity of the vaccine constructs was predicted based on their primary sequences. This assessment was conducted using the Vaxijen v2.0 server [43].

### Solubility and physicochemical properties

The solubility features of the vaccine constructs were assessed using the SOLpro server [52]. Furthermore, the various physical and chemical parameters of the vaccine constructs were calculated using the ProtParam web server [53]. The parameters evaluated included the Instability Index, Aliphatic Index, Theoretical Isoelectric Point (pI), Half-life in mammalian cellular environments, and Grand Average of Hydropathicity (GRAVY).

### Two- and three-dimensional structure prediction and model refinement

The three-dimensional (3D) structural conformation of the novel vaccine candidate was predicted using the I-TASSER server [54], an online platform for protein structure prediction. The predicted 3D structural model of the recombinant vaccine candidate was subsequently refined and enhanced using the Galaxy Refine server [55]. This web-based tool employs a robust refinement strategy that was validated during the CASP10 (Critical Assessment of Structure Prediction) experiment. The overall structural model was relaxed following the refinement process via molecular dynamics (MD) simulations [56]. This simulation process was conducted to further refine and optimize the predicted structure.

Although the secondary structure elements are evident in the three-dimensional model, the secondary structure of the multi-epitope vaccine was also predicted separately to visualize these features in a linear format. This analysis was performed using the PSIPRED 4.0 Workbench [57], available at https://bioinf.cs.ucl.ac.uk/psipred/.

### 3D model evaluation

The 3D structural model of the multi-epitope vaccine was subjected to a comprehensive evaluation using two sophisticated online analytical platforms: ProSA-web [58] and PROCHECK [59, 60]. ProSA-web, a well-established tool for assessing protein structure quality, was utilized. The scores generated by this platform were contextualized within the broader landscape of known protein structural data, thereby providing valuable insights into the model's overall quality. In addition to ProSA-web, the PROCHECK suite was used to perform an extensive stereochemical analysis of the protein structure model. This analysis provided detailed information on the model’s geometry and identified potential areas for improvement.

### Molecular docking

The potential binding affinity between the multi-epitope vaccine and TLR4 was investigated using molecular docking [61]. This process was conducted using the HDOCK server [62]. The study assessed the possible interaction between the vaccine and the receptor molecule, providing insights into its potential efficacy. For the docking process, 3D coordinates of TLR4 were utilized as the receptor structure. These coordinates were obtained from the Protein Data Bank (PDB ID: 4G8A, resolution 2.4 Å) and were employed for docking with the refined vaccine model.

Molecular docking evaluated the structural compatibility and potential interaction interfaces between the vaccine construct and TLR4. This bioinformatics approach provides a theoretical, geometry-based assessment of possible receptor–ligand interactions within a defined computational framework [63–65]. It is therefore important to emphasize that docking analyses may not directly measure binding affinity or downstream immune activation.

### Molecular dynamics (MD) simulations

The structural dynamics and conformational flexibility of the vaccine–TLR4 complex were examined through MD simulations. These simulations were carried out using the iMOD server [66]. The iMOD server employs Advanced Normal Mode Analysis (NMA) methods to provide insights into the potential conformational changes and dynamic behavior of molecular complexes. Understanding these dynamics was considered crucial, as they can significantly impact the efficacy and biological function of the complex [67].

Moreover, MD simulations were performed using Discovery Studio Client (version 16.1.0.15350) to assess the structural stability of the vaccine–TLR4 complex. The simulations ran for a total duration of 100 ns (ns) on a workstation equipped with a 64-bit operating system, 64 GB DDR5 RAM, and an Intel Core i9-13900KF 24-core processor. Key simulation parameters, including the cell geometry, target temperature, solvation model, and force field (CHARMM) [68], were configured in accordance with a previously reported protocol [69]. To monitor the conformational behavior of the vaccine–TLR4 complex throughout the simulation, root-mean-square deviation (RMSD) and root-mean-square fluctuation (RMSF) analyses of the backbone atoms were performed on the resulting MD trajectories.

### Population coverage analysis

Population coverage analysis was carried out using the IEDB population coverage tool to assess the global applicability of the selected epitopes. In this regard, the predicted human HLA alleles corresponding to the shortlisted MHC class I and MHC class II epitopes were submitted to the server separately. The tool estimates the fraction of individuals in a given population expected to present at least one epitope–HLA combination, leveraging reported HLA allele frequencies from various ethnic groups and geographic regions. Default parameters were employed for all analyses.

### Cytokine induction potential

To assess the cytokine induction potential of the selected MHC-II epitopes [70], three specialized servers were employed: IL4pred (https://webs.iiitd.edu.in/raghava/il4pred/index.php) [71] for IL-4, IL-10Pred (https://webs.iiitd.edu.in/raghava/il10pred/index.html) [72] for IL-10, and IFNepitope (https://webs.iiitd.edu.in/raghava/ifnepitope/index.php) [73] for IFN-γ. All predictions were performed with default parameters. Importantly, these analyses were performed solely for descriptive reporting and did not influence the epitope selection process.

### Reverse transcription and codon optimization

Following reverse translation of the recombinant vaccine sequence by the online SMS server [74], the Vector Builder tool (available at https://en.vectorbuilder.com/tool/codon-optimization.html) was employed to assess the GC content and Codon Adaptation Index (CAI) values of the nucleotide sequence for the *E. coli* K12 strain.

### B-cell epitope prediction

To further support the antigenic potential of the designed multi-epitope vaccine, discontinuous B-cell epitopes were predicted using the ElliPro server [75], a tool available within the IEDB database. This algorithm predicts B-cell epitopes based on flexibility and solvent accessibility. Predictions were performed using default parameters, including a minimum score threshold of 0.5 and a maximum distance of 6 Å.

### In silico immune simulation

The immunogenicity and immune response profile of the designed vaccine were analyzed via in silico immune simulation using the C-ImmSim server, available at https://kraken.iac.rm.cnr.it/C-IMMSIM/ [76], with default parameters.

### Disulfide engineering analysis

Disulfide bonds are thought to decrease conformational entropy and raise the free energy of the denatured state, thereby enhancing the stability of the folded protein conformation [77]. However, not all engineered disulfides result in increased stability [78]. In this study, in silico disulfide engineering analysis of the designed multi-epitope vaccine construct was performed to assess its structural stability using Disulfide by Design 2.0 [78], available at http://cptweb.cpt.wayne.edu/DbD2/.

## Results

### Top-ranked T-cell epitopes

Potential epitope regions within the SART3 antigen were identified using the IEDB platform. These regions were evaluated for their binding capacity to human and murine MHC-I molecules (with lengths of 9 and 10 amino acids) (Supplementary data 3) and MHC-II molecules (spanning 14 and 15 amino acids) (Supplementary data 4). For MHC-I binding, three epitope sequences (NPDFKVFRY, RNCPWTVALW, and YPEHVCEVL) were determined to be up-and-coming candidates based on established criteria. Among the experimentally validated MHC-I epitopes, RLEKVHSLFR, RLAEYQAYI, and HVYDLFEKA were identified as the most favorable due to their desirable attributes (Table 1). In the case of MHC-II binding, four epitope sequences (VKDLRLVTNRAGKPK, FKVFRYSTSLEKHK, KVFRYSTSLEKHKL, and EYAMASSAESSPGE) were recognized as potential candidates based on prediction criteria. The most favorable experimentally validated MHC-II epitopes were KPKGLAYVEYENES and ESVIQNYNKALQQL, as shown in Table 2.

**Table 1:** Candidate CD8 + epitopes

							Docking scores with different alleles in human and mice (kcal/mol)					
MHC-I alleles with percentile rank score < 1	Allele, with the most favorable percentile rank score	Percentile rank	Start	Epitope	Vaxijen score	Experimentally validated? (yes/no)	HLA—A0201	HLA—B3501	H2-Kd	H2-Ld	H2-Dd	Binding to the MHC-I in Mice, based on percentile rank scores from IEDB? (score < 1, Yes/No)
HLA-B*35:01	HLA-B*35:01	0.04	6	NPDFKVFRY	1.4203	No	− 260.257	− 238.174	− 205.323	− 303.361	− 200.887	Yes
HLA-A*01:01												
HLA-B*18:01												
HLA-B*15:02												
HLA-A*26:01												
HLA-B*08:01												
HLA-B*58:01	HLA-B*58:01	0.33	13	RNCPWTVALW	1.3665	No	− 273.344	− 278.704	− 237.68	− 309.024	− 252.758	Yes
HLA-B*35:01	HLA-B*35:01	0.17	1	YPEHVCEVL	1.0875	No	− 249.004	− 215.23	− 222.621	− 246.666	− 203.525	Yes
HLA-B*51:01												
HLA-B*07:02												
HLA-A*31:01	HLA-A*31:01	0.25	3	RLEKVHSLFR	1.49	Yes	− 237.771	− 206.322	− 222.85	− 210.332	− 202.376	No
HLA-A*03:01												
HLA-A*02:01	HLA-A*02:01	0.03	9	RLAEYQAYI	1.2136	Yes	− 244.719	− 214.617	− 220.832	− 234.478	− 214.257	No
HLA-B*52:01												
HLA-A*02:01	HLA-A*02:01	0.65	48	HVYDLFEKA	1.16	Yes	− 218.606	− 211.824	− 213.619	− 255.38	− 200.827	No
HLA-A*26:01

**Table 2:** Candidate CD4 + epitopes

							Docking scores with different alleles in humans and mice (kcal/mol)					
MHC-II alleles with percentile rank score < 1	Allele, with the most favorable percentile rank score	Percentile rank	Start	Epitope	Vaxijen score	Experimentally validated? (yes/no)	HLA-DRB1_1101	H2-IAd	Binding to the MHC-I in Mice, based on percentile rank scores from IEDB? (score < 1, yes/no)	IL-4 inducer	IL-10 inducer	IFN-γ inducer
HLA-DRB1*04:01	HLA-DRB1*04:01	0.58	827	VKDLRLVTNRAGKPK	1.2446	No	− 230.66	− 239.709	Yes	Non-inducer	Non-inducer	Negative
HLA-DRB1*16:02												
HLA-DRB1*15:01												
HLA-DRB1*13:01	HLA-DPA1*02:01/DPB1*05:01	0.09	789	FKVFRYSTSLEKHK	0.8421	No	− 285.003	− 271.556	Yes	Non-inducer	Inducer	Negative
HLA-DPA1*02:01/DPB1*05:01												
HLA-DPA1*02:01/DPB1*05:01												
HLA-DPA1*02:01/DPB1*05:01	HLA-DPA1*02:01/DPB1*05:01	0.19	790	KVFRYSTSLEKHKL	0.595	No	− 264.157	− 240.115	Yes	Non-inducer	Inducer	Negative
HLA-DQA1*03:03/DQB1*03:01	HLA-DQA1*03:03/DQB1*03:01	0.65	69	EYAMASSAESSPGE	0.5771	No	− 228.639	− 200.305	Yes	Non-inducer	Non-inducer	Negative
HLA-DPA1*02:01/DPB1*05:01	HLA-DPA1*02:01/DPB1*05:01	0.14	839	KPKGLAYVEYENES	0.642	Yes	− 217.958	− 225.755	No	Inducer	Non-inducer	Negative
HLA-DPA1*01:03/DPB1*04:02												
HLA-DRB1*13:01	HLA-DRB1*13:01	0.64	279	ESVIQNYNKALQQL	0.8048	Yes	− 214.031	− 224.714	No	Inducer	Non-inducer	Negative

### Vaccine engineering

The multi-epitope vaccine construct was designed to encompass the most immunogenic epitopes of the SART3 protein. Six epitopes with high binding affinity for MHC class I molecules were selected for inclusion, along with six additional epitopes predicted to bind MHC class II molecules with high affinity. A peptide adjuvant was strategically placed at the N-terminus to enhance immunogenicity. Furthermore, a histidine tag was added to the C-terminus to facilitate purification of the recombinant vaccine. A diverse array of 110 distinct vaccine constructs was produced, each consisting of 344 amino acid residues (Supplementary data 5).

### Allergenicity, antigenicity, physicochemical properties, and solubility of the vaccine candidate

The library of 110 multi-epitope vaccine candidates underwent extensive screening. This comprehensive evaluation assessed each construct’s allergenicity profile, predicted antigenic properties, physicochemical solubility parameters, and other pertinent biophysical characteristics (Supplementary data 6).

**Fig. 2:**
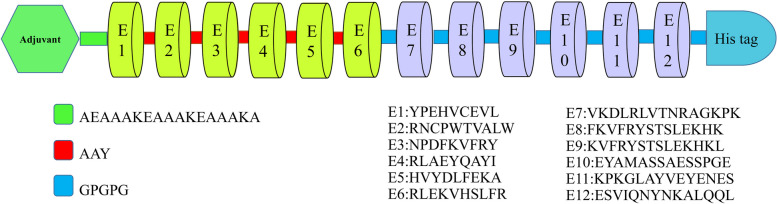
Schematic view of the candidate vaccine elements

The most promising vaccine molecule design was identified and selected as the final optimized sequence through a meticulous analysis based on quantitative scoring of critical properties—a desirable combination of traits characterized this lead candidate. Non-allergenicity was confirmed for the construct. An excellent computed instability index of 17.16 was observed. A favorable aliphatic index value of 76.48 was determined. The theoretical pI was calculated to be 6.62. In mammalian reticulocyte cells, a predicted half-life of 30 h was estimated. A GRAVY score of -0.315 was computed. An antigenic Vaxijen score of 0.67 was obtained. Additionally, a probability of 0.96 for favorable aqueous solubility was calculated. Figure 2 presents a schematic illustration of the linear organization of the optimized multi-epitope vaccine constructs.

### Modeling, structural refinement, and evaluation

**Fig. 3:**
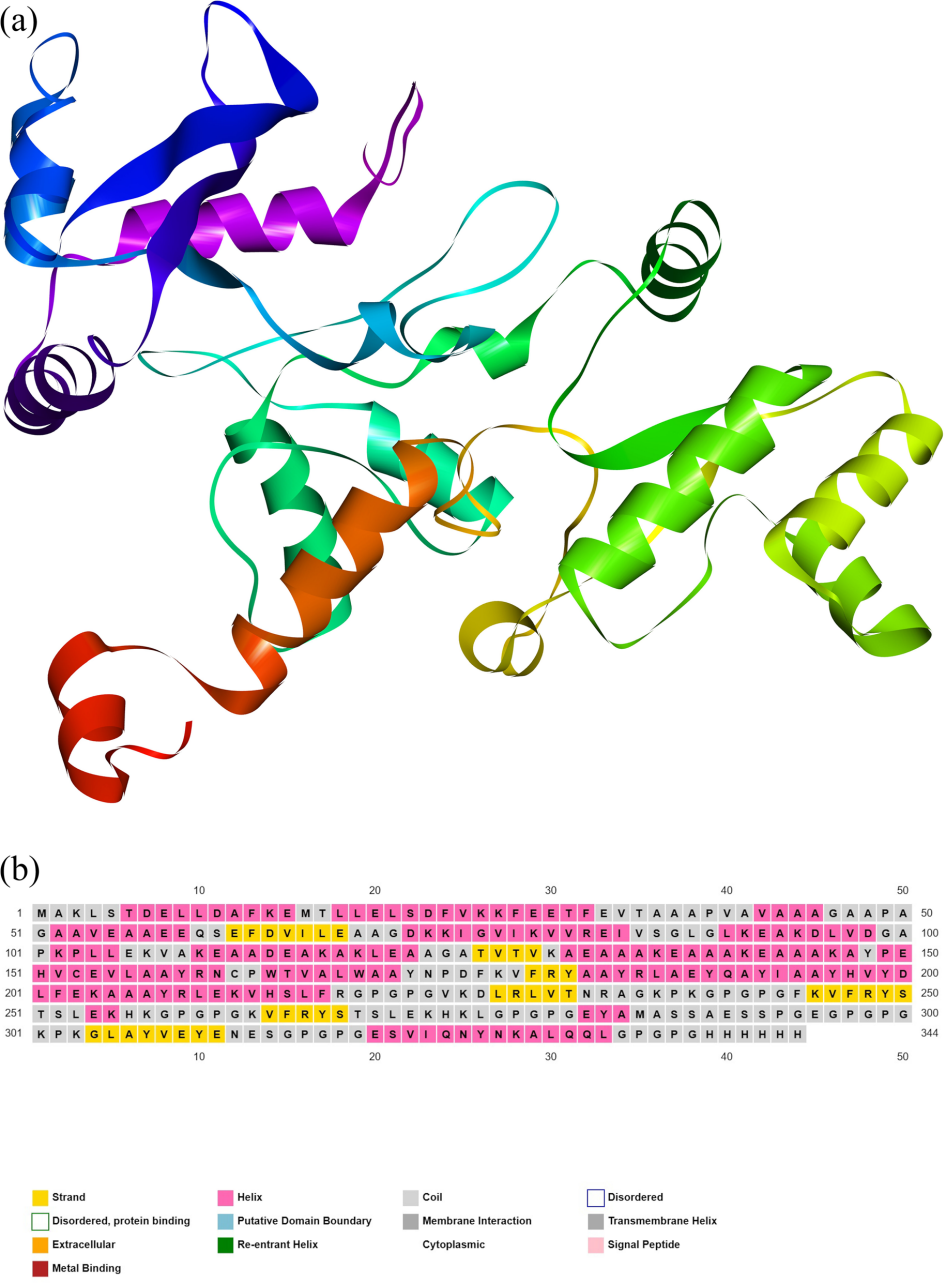
**a** Three-dimensional structure of the recombinant multi-epitope vaccine, depicted with a color gradient along the polypeptide chain. The N-terminus, encompassing the adjuvant moiety, is shown in blue, transitioning to green at the C-terminus. **b** Predicted secondary structure of the vaccine. According to the predictions, the secondary structure of the multi-epitope construct consisted of approximately 49.13% alpha-helices, 11.05% beta-sheets, and 39.83% coils.

The vaccine's 3D structure was predicted in silico using the I-TASSER server. The vaccine model earned a C-score of − 3.34, a reliability metric ranging from − 5 to 2, with higher values indicating greater confidence in the model’s accuracy. Subsequently, the 3D model was refined using the GalaxyRefine tool and further validated. The refined model is presented in Fig. 3a, and the predicted secondary structure of the multi-epitope vaccine is shown in Fig. 3b.

**Fig. 4:**
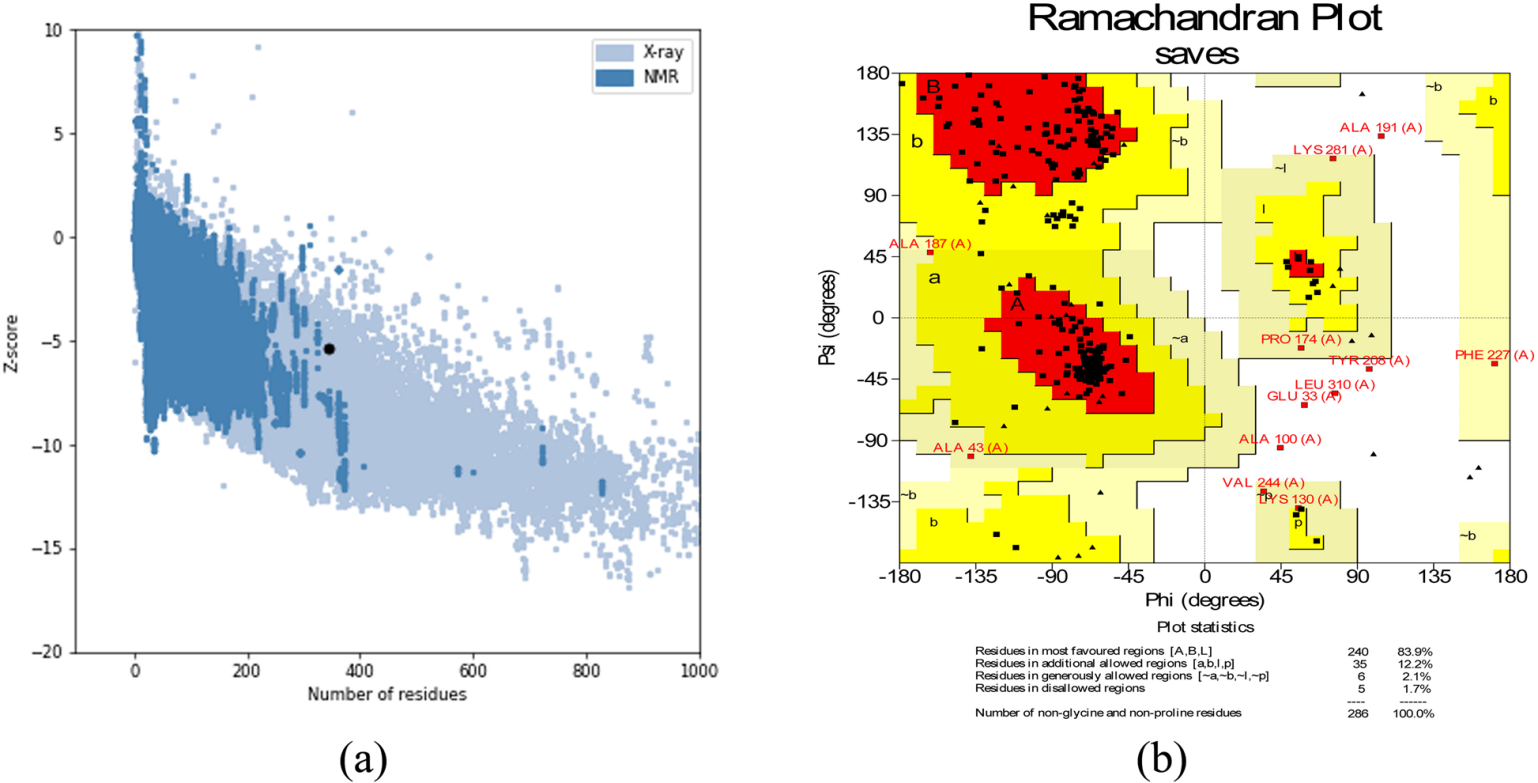
The refined three-dimensional model of the vaccine construct was evaluated using two methods: **a** ProSA-web analysis and **b** Ramachandran plot analysis.

ProSA-web analysis indicated a *Z*-score of − 5.37 for the refined model, a value consistent with native proteins (Fig. 4a). Furthermore, Ramachandran plot analysis from the PROCHECK server revealed that 83.9% of residues resided in the most favored regions, with 12.2% in additionally allowed regions, 2.1% in generously allowed regions, and 1.7% in the disallowed areas (Fig. 4b).

### Protein–protein docking between the vaccine and TLR4

In silico analysis of the vaccine candidate's binding affinity to TLR4 was performed using the HDOCK server. The most favorable binding mode between the multi-epitope vaccine and TLR4 was selected based on the lowest Gibbs free energy of binding (− 265.61 kcal/mol) and a high confidence score of 91%.

### MD analysis

**Fig. 5:**
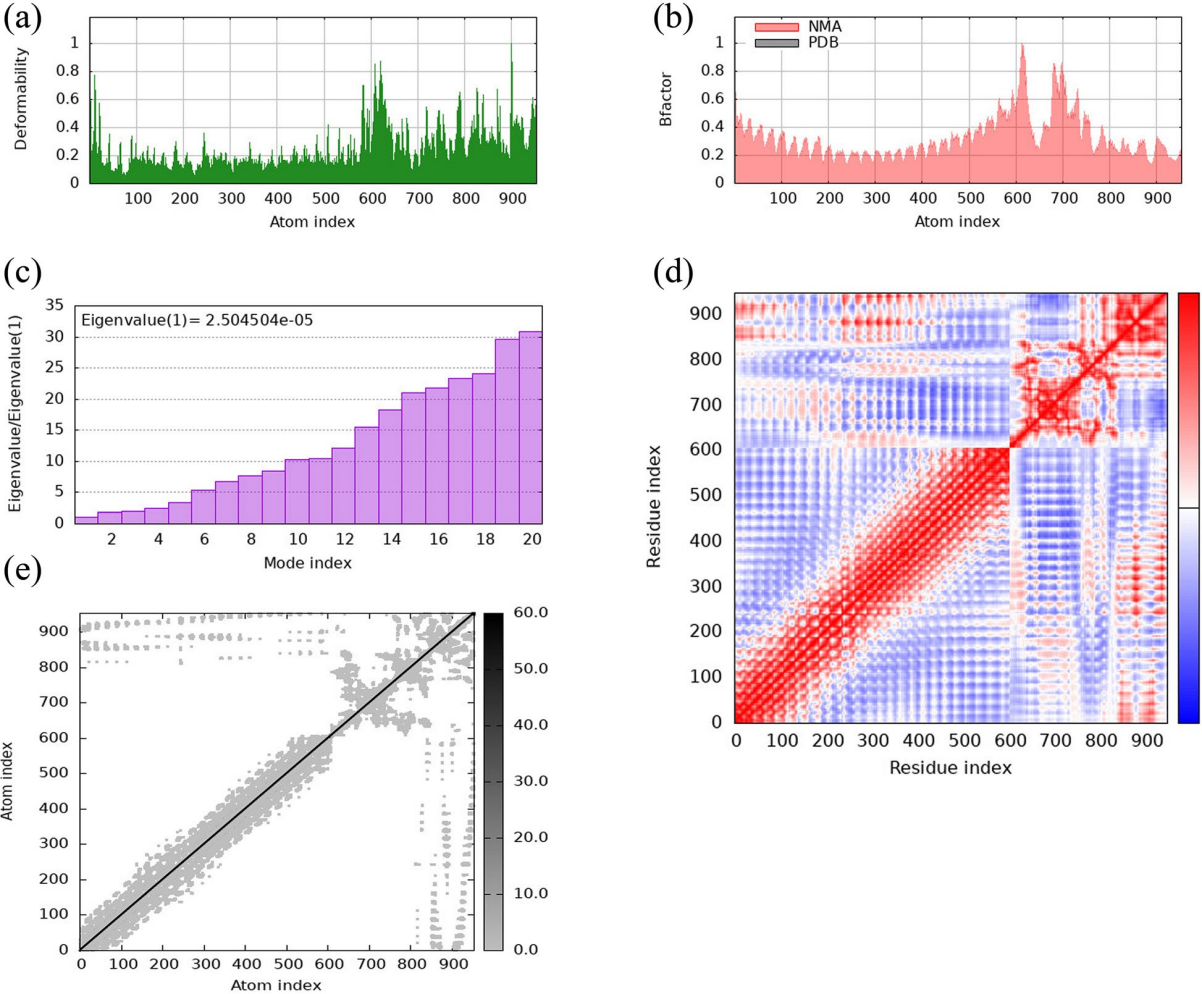
In silico deformability analysis performed on the vaccine–TLR4 complex using the iMODS server. These analyses included **a** a visual representation of deformability characteristics, **b** a plot of B-factor values, **c** an eigenvalue assessment of the complex's intrinsic dynamics, **d** a covariance matrix visualization of correlated motions between residues (red: concerted, white: uncorrelated, blue: anti-correlated), and **e** an elastic network model depicting relative inter-residue rigidity (darker gray: more rigid). TLR4, Toll-like receptor 4

In silico evaluation of the vaccine–TLR4 complex stability using the iMOD server suggested minimal structural distortion. Figure 5a visually corroborates this observation, depicting a limited number of hinges indicative of restricted flexibility within the complex. Furthermore, the B-factor plot in Fig. 5b reinforces this notion, with low root-mean-square fluctuation values indicating high structural stability. Additionally, the eigenvalue of 2.5 × 10^−5^ in Fig. 5c indicates a substantial energetic barrier to deforming the vaccine bound to TLR4, underscoring its rigid conformation. The covariance matrix in Fig. 5d shows correlations between amino acid pairs, with red indicating positive correlations, white indicating no correlation, and blue indicating anti-correlations. This information can be used to identify potential interaction hotspots within the complex. Finally, the elastic network model visualization in Fig. 5e highlights regions with varying degrees of flexibility. Darker gray shading indicates stiffer, more rigid protein segments within the complex.

**Fig. 6:**
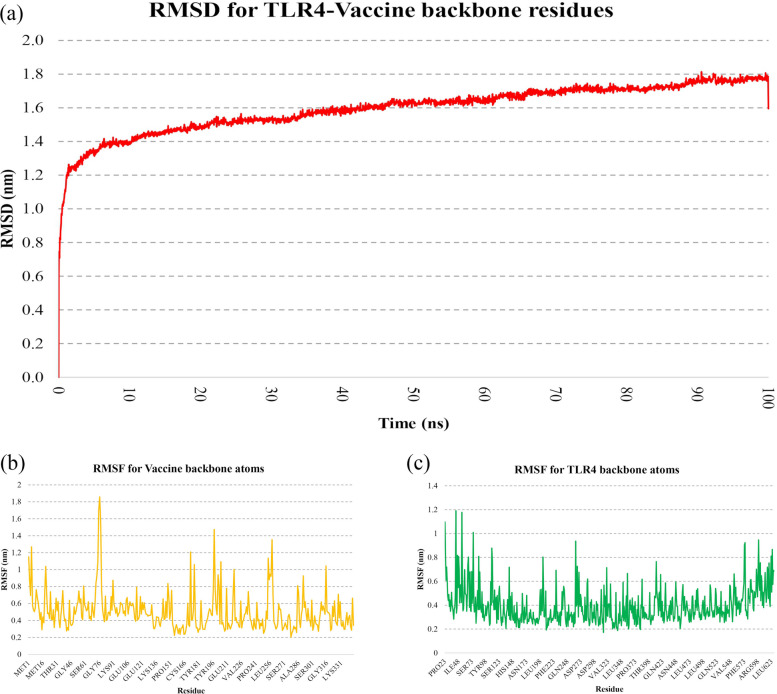
Conformational stability and residue flexibility of the vaccine–TLR4 complex during MD simulation. **a** Backbone root mean square deviation plotted over 100 ns. Root mean square fluctuation profiles for **b** vaccine and **c** TLR4 backbone atoms within the docked complex. TLR4, Toll-like receptor 4; MD, molecular dynamics

**Fig. 7:**
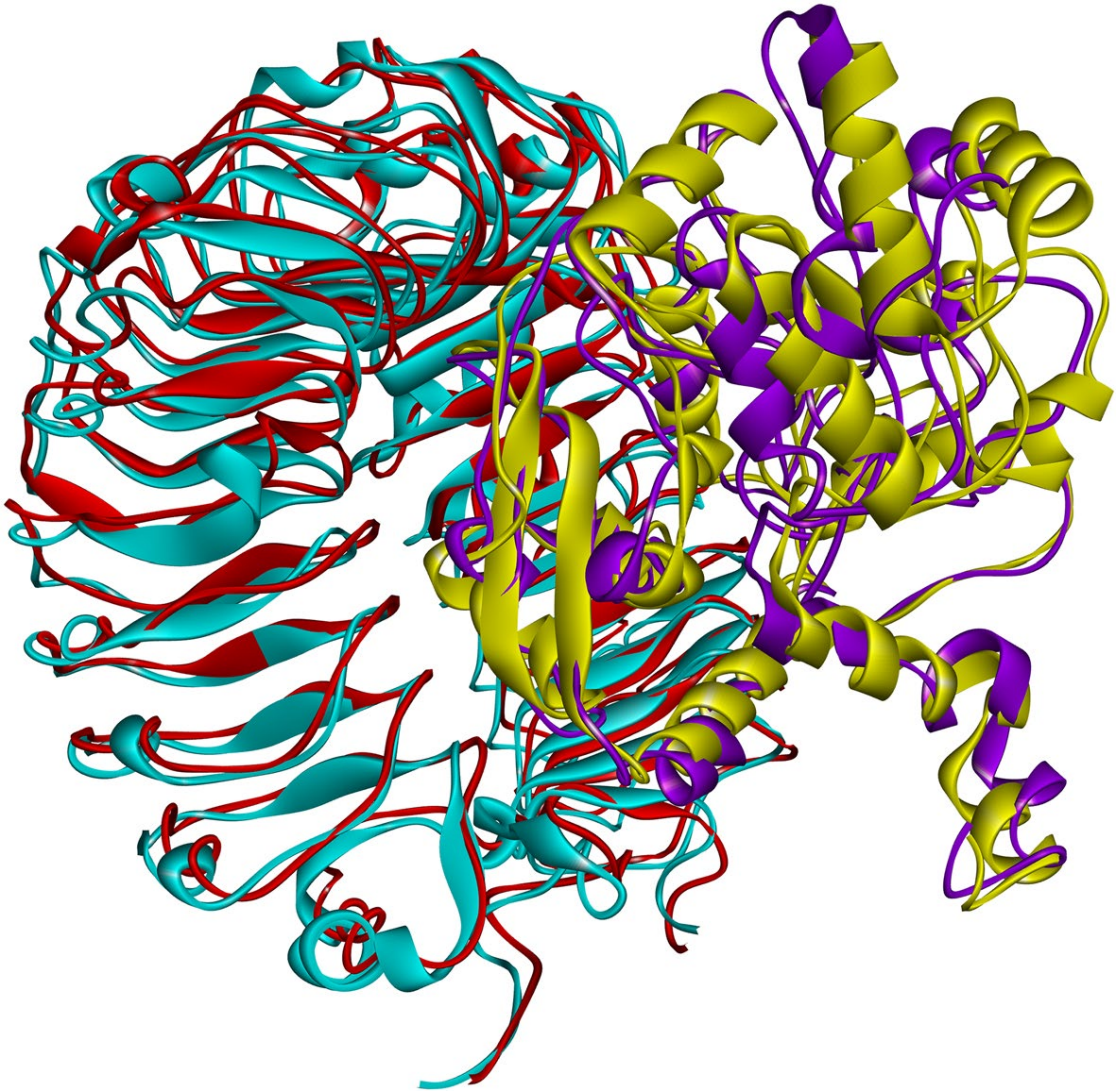
Conformational comparison of the vaccine–TLR4 complex before and after 100 ns MD simulation. The initial (pre-simulation) and final (post-simulation) structures are superimposed, with the vaccine colored yellow and purple, respectively, and TLR4 colored cyan and red, respectively. TLR4, Toll-like receptor 4; MD, molecular dynamics

**Fig. 8:**
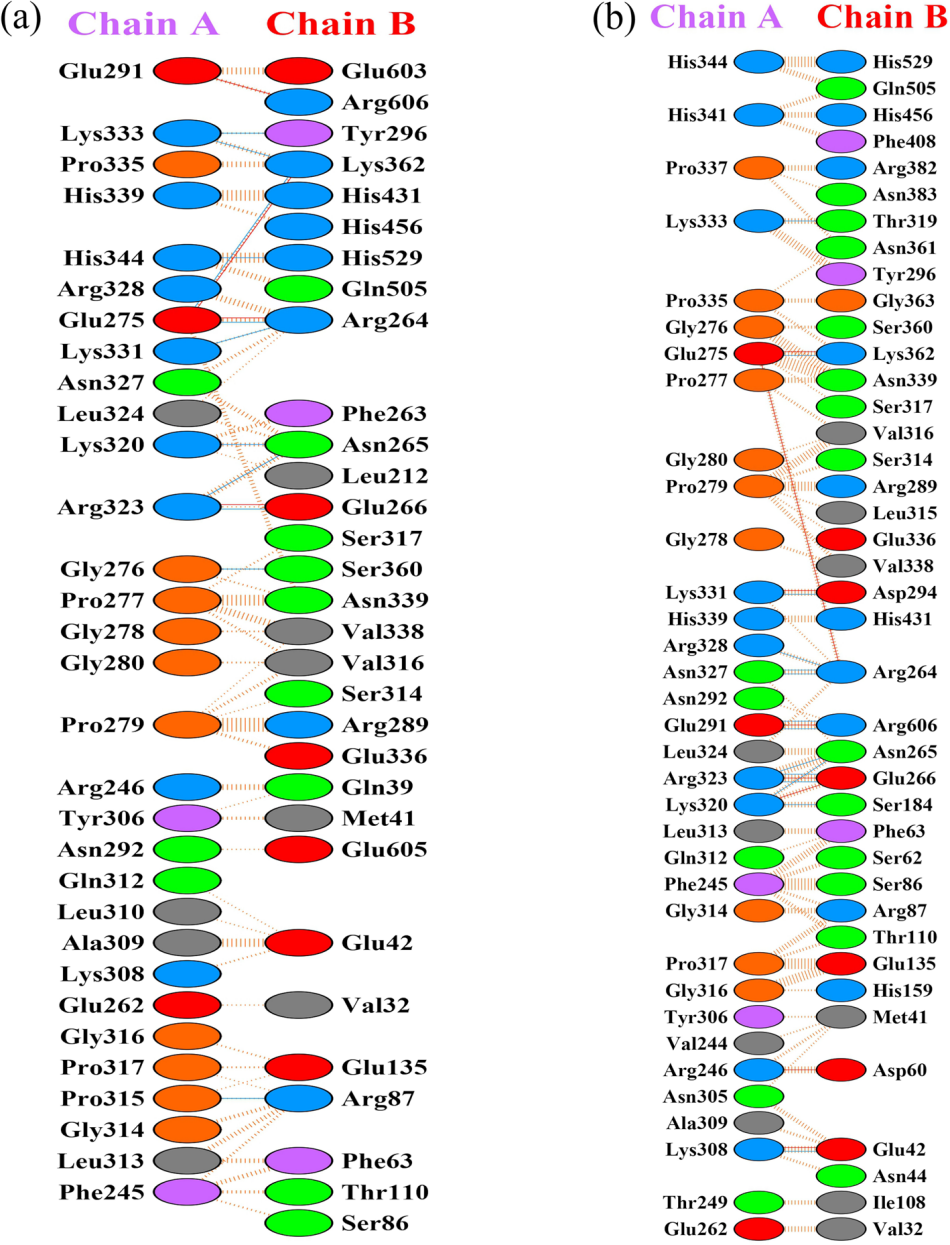
Comparison of vaccine–TLR4 binding interfaces before and after MD simulation. PDBsum was used to visualize interactions in the initial structure (**a**) and following 100 ns of simulation (**b**), with hydrogen bonds depicted as blue lines. TLR4, Toll-like receptor 4

The RMSD of the vaccine–TLR4 complex increased rapidly at the simulation outset, reaching ~ 1.4 nm by 10 ns, followed by minor fluctuations and a final RMSD of 1.8 nm at 100 ns (Fig. 6a). RMSF analysis showed backbone fluctuations ranging from 0.2 to 1.9 nm for the vaccine and 0.2–1.2 nm for TLR4 (Fig. 6b, c). Pre- and post-simulation structures were superimposed using Discovery Studio Visualizer to visualize conformational changes over 100 ns (Fig. 7). Residue-level interactions between the vaccine and TLR4 were further analyzed using the PDBsum server, enabling comparison of binding interfaces before and after MD simulation (Fig. 8). A detailed summary of hydrogen bond interactions is provided in Table 3.

**Table 3:** Intermolecular hydrogen bonds between the multi-epitope vaccine and TLR4

A, Before MD simulation		
Vaccine	TLR4	Bond length (Å)
PRO315	ARG87	3.23
GLU275	ARG264	2.66
LYS331	ARG264	2.99
ARG323	ASN265	3.25
LYS320	ASN265	2.90
ARG323	GLU266	3.07
LYS333	TYR296	3.07
GLY276	SER360	3.25
GLU275	LYS362	2.92
LYS333	LYS362	2.34
HIS344	HIS529	2.35
B, After MD simulation		
Vaccine	TLR4	Bond length (Å)
LYS308	GLU42	2.43
LYS320	SER184	2.66
ASN327	ARG264	2.85
ASN327	ARG264	3.11
ARG328	ARG264	2.83
ARG323*	ASN265	2.71
LYS320*	ASN265	3.29
ARG323*	GLU266	2.97
ARG323	GLU266	2.75
LYS331	ASP294	2.60
LYS333	THR319	2.47
GLU275*	LYS362	3.05
GLU291	ARG606	3.10
GLU291	ARG606	2.90

Distances are reported for the docked complex, with asterisks (*) marking residues that sustained stable interactions over the course of the molecular dynamics simulation

### Population coverage of selected epitopes

The selected MHC class I epitopes demonstrated an estimated population coverage of 84.72%**,** indicating broad applicability across diverse human populations. The average number of epitope–HLA class I hits was 1.93**,** with 90% of the population predicted to recognize at least 0.65 epitope–HLA combinations (PC90)**.** In contrast, the selected MHC class II epitopes exhibited a population coverage of 37.46%, with an average of 0.49 epitope–HLA class II hits and a PC90 value of 0.16.

### IL-4, IL10, and IFN-γ induction

Among the six top-ranked MHC-II epitopes selected for inclusion in the multi-epitope vaccine construct, two epitopes, KPKGLAYVEYENES and ESVIQNYNKALQQL, were predicted to induce IL-4. Additionally, two other epitopes, FKVFRYSTSLEKHK and KVFRYSTSLEKHKL, were found to be capable of inducing IL-10. However, according to the prediction algorithms, none of the six epitopes was predicted to induce IFN-γ (Table 2).

### Reverse transcription and codon optimization

**Fig. 9:**
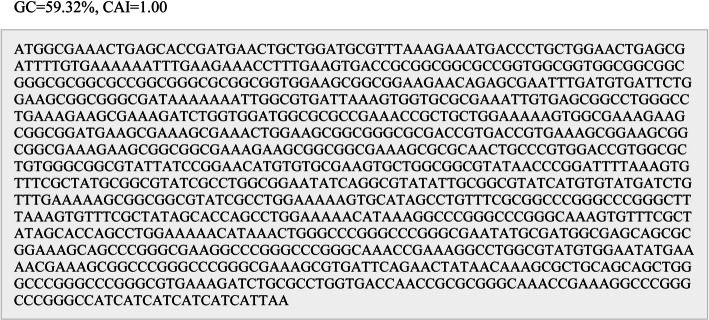
The nucleotide sequence encoding the recombinant multi-epitope vaccine construct

Following reverse transcription facilitated by the SMS server, the nucleotide sequence of the multi-epitope vaccine was analyzed using the Vector Builder tool to determine its Codon Adaptation Index (CAI). The sequence exhibited a favorable CAI value of 1.0 for the *E. coli* K12 strain and a GC content of 59.32% (Fig. 9).

### Discontinuous B-cell epitopes

**Fig. 10:**
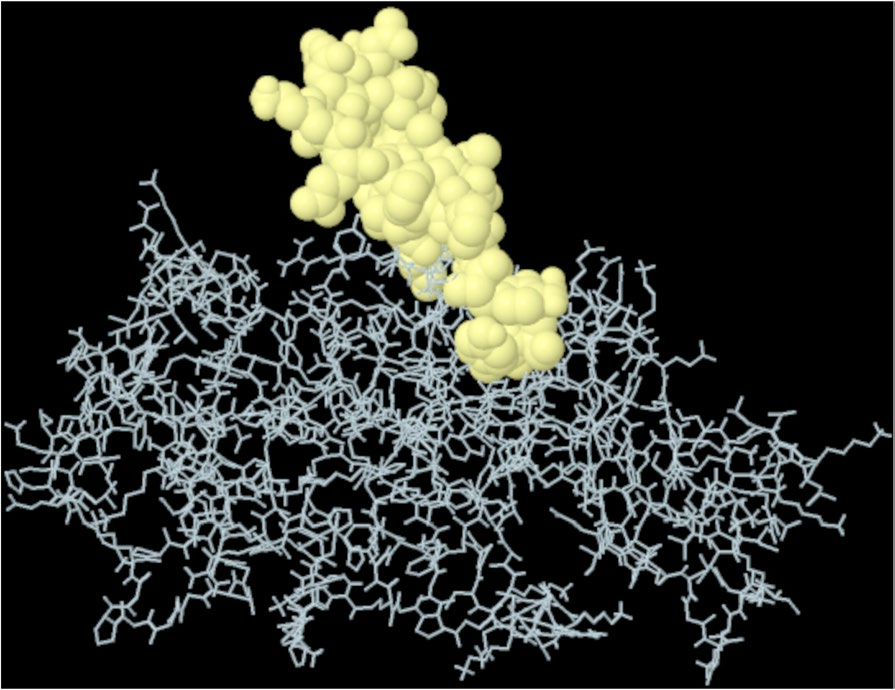
The highest-scoring discontinuous B-cell epitope in the multi-epitope vaccine (32 residues; score 0.765)

A total of 11 discontinuous B-cell epitopes were identified within the multi-epitope vaccine using ElliPro (Table 4). The predominant epitope, comprising 32 residues, exhibited a score of 0.765 (Fig. 10).

**Table 4:** Discontinuous B-cell epitopes in the multi-epitope vaccine predicted by ElliPro

Residues	Number of residues	Score
A:M1, A:A2, A:K3, A:L4, A:S5, A:T6, A:D7, A:E8, A:L9, A:L10, A:D11, A:A12, A:F13, A:K14, A:E15, A:M16, A:T17, A:L18, A:L19, A:E20, A:L21, A:S22, A:D23, A:K26, A:Y172, A:N173, A:P174, A:D175, A:F176, A:K177, A:Y189, A:Q190	32	0.765
A:L67, A:E68, A:A69, A:A70, A:G71, A:D72, A:K73, A:K74, A:I75, A:G76, A:V77, A:I78, A:K79, A:V80, A:V81, A:R82, A:E83, A:I84, A:V85, A:S86, A:G87, A:L88, A:G89, A:L90, A:K91, A:E92, A:A93, A:K94, A:D95, A:L96, A:V97, A:D98, A:G99, A:A100, A:P101, A:K102, A:P103, A:L104, A:E115, A:A118, A:K119, A:A122, A:A123, A:G124, A:A125, A:T126	46	0.745
A:A207, A:K308, A:A309, A:L310, A:Q311, A:Q312, A:L313, A:G314, A:P315, A:G316, A:P317, A:G318, A:V319, A:K320, A:R323	15	0.734
A:G242, A:K243, A:V244, A:F245, A:S248, A:T249, A:S250, A:L251, A:E252, A:H254, A:K255	11	0.728
A:P273, A:G274, A:E275, A:G276, A:P277, A:G278, A:P279, A:G280, A:K281, A:P282, A:L324, A:N327, A:R328, A:G330, A:K331, A:P332, A:K333	17	0.727
A:K253, A:L256, A:G257, A:P258, A:G259, A:P260, A:G261, A:E262, A:Y263, A:A264, A:M265, A:A266, A:S267, A:S268, A:A269, A:E291, A:N292, A:E293, A:S294, A:G295, A:G297, A:P298, A:S301	23	0.698
A:A135, A:K136, A:E137, A:A138, A:K141	5	0.627
A:G334, A:P335, A:G336, A:P337, A:G338, A:H339, A:H340, A:H341, A:H342, A:H344	10	0.613
A:K204, A:A205, A:A206	3	0.591
A:E142, A:A145, A:K146, A:A147, A:N149, A:C150	6	0.544
A:A50, A:G51, A:A52, A:A53, A:V54, A:E55, A:E59, A:Q60, A:E62, A:L105, A:E106, A:K107, A:V108	13	0.541

### Vaccine immune simulation

**Fig. 11:**
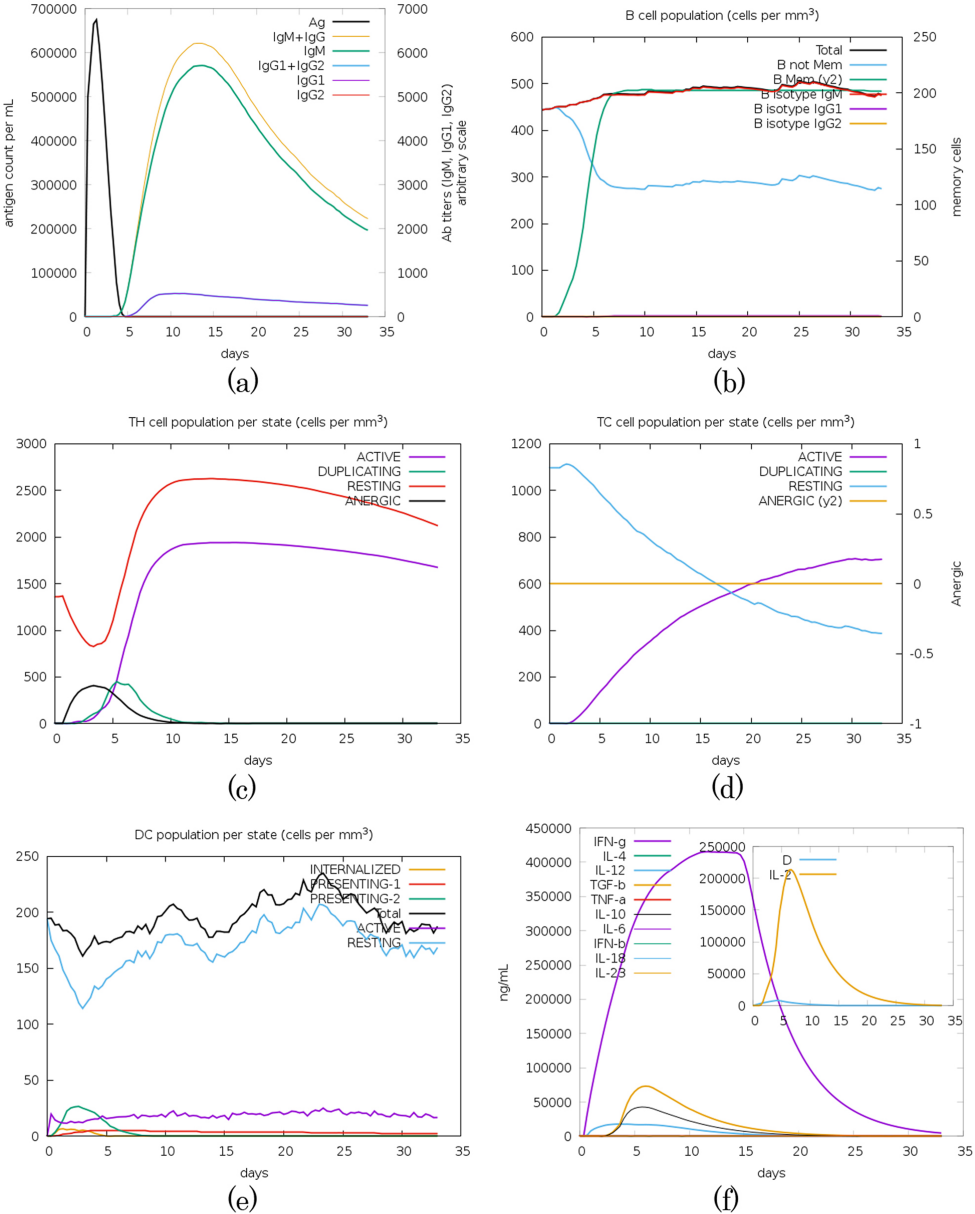
Time-course analysis of immune responses over 35 days post-immunization. The panels show **a** antibody titers, **b** B-cell populations, **c** T helper cell subsets, **d** cytotoxic T cell states, **e** dendritic cell populations, and **f** cytokine production

The in silico immune simulations demonstrated a robust immune response following antigen administration. As shown in Fig. 11a, the antigen (represented by the black line) rapidly decreased from an initial peak of approximately 700,000 counts per mL at day 2, dropping to near-zero levels by day 5. This clearance coincided with a significant rise in antibody titers, particularly IgM (green line) and the combined IgM + IgG response (yellow line), which reached peak concentrations of approximately 5500 and 6000 (arbitrary units), respectively, between days 10 and 15—indicative of successful humoral immunity. A subsequent sustained elevation in IgG1 (purple line) was also observed.

Figure 11b further illustrates the dynamics of the B cell population, revealing an initial expansion and rapid phenotypic shift. Although the total B cell population remained relatively stable at approximately 500 cells/mm^3^, a sharp decline in non-memory B cells (blue line) was observed, which closely correlated with a rapid differentiation into memory B cells (green line). Memory B cells peaked at over 200 cells/mm^3^ by day 6. In addition, IgG1- and IgG2-isotype B cells emerged and stabilized at low but persistent levels, confirming the induction of a sustained class-switched adaptive humoral response.

Concurrently, T helper (TH) cells exhibited a strong activation and proliferation phase (Fig. 11c). The resting TH population initially decreased from approximately 1350 cells/mm^3^ before recovering. In comparison, active TH cells rose sharply to nearly 1900 cells/mm^3^ by day 8 and remained elevated at around 1600 cells/mm^3^ through day 32. The proliferating TH population peaked at approximately 2600 cells/mm^3^ around day 5, then gradually declined, reflecting the coordinated expansion essential for orchestrating both humoral and cellular immunity. A similar pattern was observed for anergic TH cells, with a peak at day 3.

Cytotoxic T (TC) cells also demonstrated a clear activation profile (Fig. 11d). Beginning around day 2, active TC cells (purple line) exhibited a steady, gradual increase, reaching approximately 700 cells/mm^3^ by day 30. Concurrently, the resting TC cell population also declined.

Dendritic cell (DC) populations exhibited a notable initial reduction, with resting DCs declining from approximately 185 cells/mm^3^ to roughly 130 cells/mm^3^ by day 3 (Fig. 11e). This early decrease is consistent with DC activation and antigen internalization. Subsequently, the total DC count recovered and stabilized between 190 and 210 cells/mm^3^ from day 15 onward. Throughout the simulation, the Presenting-1, Presenting-2, and Internalized DC subpopulations remained at low levels, while active DCs maintained a steady baseline. This pattern indicates a transition from an early activation phase to a steady-state antigen-presenting equilibrium.

Finally, the cytokine profile (Fig. 11f) revealed transient upregulation of key pro-inflammatory and regulatory cytokines. A pronounced induction of IFN-γ (purple line) was observed, peaking at over 400,000 ng/mL around day 12 before gradually resolving. The inset graph further highlights an early, sharp spike in IL-2 (yellow line), reaching approximately 200,000 ng/mL by day 6. The timely secretion and subsequent decline of factors such as IFN-γ and IL-2 indicate a well-orchestrated immune activation followed by appropriate resolution.

### Disulfide bond engineering

Disulfide engineering analysis was conducted using the Disulfide by Design 2 server to enhance the structural stability of the modeled protein. The analysis predicted 26 residue pairs as potential sites for disulfide bond formation upon cysteine substitution (Table 5). These candidates exhibited a wide range of predicted *χ*^3^ dihedral angles and bond energies, with calculated values spanning 1.16–8.16 kcal/mol. The Val80–Ala123 pair exhibited the lowest bond energy (1.16 kcal/mol), indicating the optimal geometry for disulfide bond formation. The Glu68–Thr126 pair also showed a comparatively low energy (2.01 kcal/mol), indicating a potentially viable configuration.

**Table 5:** Potential residue pairs for disulfide bond engineering and their geometric parameters

Residue 1		Residue 2		Bond information	
Residue number	Residue name	Residue number	Residue name	Chi3	Energy (kcal/mol)
5	SER	8	GLU	− 98.85	3.49
31	THR	36	ALA	− 89.05	4.35
37	ALA	41	ALA	104.88	2.57
48	ALA	57	ALA	− 65.98	4
54	VAL	57	ALA	− 102.4	6.11
62	GLU	107	LYS	− 86.53	2.89
63	PHE	113	ALA	84.01	5.53
64	ASP	103	PRO	− 108.4	2.89
65	VAL	104	LEU	− 114.3	4.46
68	GLU	126	THR	118.37	2.01
80	VAL	123	ALA	− 89.07	1.16
85	VAL	88	LEU	− 108.8	2.66
88	LEU	92	GLU	− 86.95	2.52
99	GLY	102	LYS	71.48	4.34
108	VAL	112	ALA	99.74	4.14
120	LEU	127	VAL	− 73.55	7.47
135	ALA	139	ALA	101.8	3.95
155	ALA	216	LEU	− 78.16	3.64
162	PRO	185	ARG	109.32	5.5
166	CYS	178	VAL	− 106.2	4.42
189	TYR	195	ALA	− 63.54	8
206	ALA	209	ARG	119.05	4.1
217	PHE	219	GLY	125.19	4.89
267	SER	291	GLU	− 91.52	8.16
268	SER	289	GLU	103.87	3.83
273	PRO	276	GLY	− 82.19	2.94

## Discussion

SART3, p110, or Tip110, plays a critical role in the spliceosome machinery. It facilitates the assembly and disassembly of the U4/U6 small nuclear ribonucleoprotein (snRNP) complex, a vital step for pre-mRNA splicing. This process ensures the generation of mature mRNA transcripts. Beyond its established role, accumulating evidence suggests SART3 acts as a multifaceted regulator within the spliceosome. Its interactions extend to influence DNA repair pathways, developmental processes, cancer progression, and cellular responses to environmental stresses [2, 14, 17, 79, 80].

The potential for SART3-based immunotherapy offers promising avenues for cancer treatment. Studies have identified SART3 antigen and its derived peptides as potential targets for specific immunotherapy in a significant portion of breast (HLA-A24 +) [20] and colorectal (HLA-A24 +) cancer [8] patients. Additionally, research suggests that specific SART3 peptides, like SART3(511–19) and SART3(734–42), hold promise for peptide-based immunotherapy in HLA-A3 supertype + prostate cancer patients [24]. Furthermore, Mohamed et al. [81] propose the SART3(109–118) peptide as a potential cancer vaccine candidate, not only for HLA-A24 + individuals but also for those with HLA-A11 +, HLA-A31 +, and HLA-A33 + in the context of prostate cancer treatment.

T-cell-mediated cytotoxicity, a cornerstone of antitumor immunity, has driven significant research in cancer immunotherapy. CD8+ cytotoxic T lymphocytes (CTLs) play a particularly crucial role by directly eliminating cancer cells through mechanisms like perforin and granzyme release [82]. Given the promising potential of SART3-based immunotherapy, this study sought to develop a novel multi-epitope vaccine. This study performed advanced immunoinformatics tools to identify and integrate T cell epitopes derived from SART3.

In addition to tumor-associated antigens, the vaccine design incorporates a TLR4 agonist as an adjuvant to potentiate the immune response further. TLR4 was prioritized over other commonly used TLR agonists—such as TLR2, TLR3, and TLR9—for several reasons: (i) TLR4 agonists are clinically advanced and known to induce robust Th1-polarized and CD8⁺ T cell responses, which are optimal for cytotoxic vaccine platforms; (ii) the 50S ribosomal protein L7/L12 from *Mycobacterium tuberculosis* specifically engages TLR4 to enhance T cell responses; and (iii) incorporating a single defined TLR4 agonist offers a tractable in silico modeling framework, with future studies planned to compare TLR4, TLR3, and TLR9-based versions in vivo to optimize SART3 vaccination [83, 84].

TLR4 activation on antigen-presenting cells potently enhances antitumor immunity by promoting dendritic cell maturation, upregulating costimulatory molecules, and driving Th1-polarized T cell responses. Numerous preclinical studies have demonstrated that TLR4 agonists synergize with cancer vaccines to induce durable tumor control. However, TLR4 signaling has also been implicated in tumor-promoting inflammation and immune evasion in certain cancer models, underscoring its context-dependent effects. We therefore emphasize that while our TLR4 agonist is designed to boost cross-priming and effector T cell function, its net impact ultimately requires empirical evaluation in vivo [83, 85–87].

Supporting this approach, multiple immunoinformatics studies have incorporated the 50S ribosomal protein L7/L12 as an adjuvant in multi-epitope vaccine designs. These investigations have consistently demonstrated, through computational predictions, that the protein is non-toxic [88], contributes to the favorable solubility and stability of the vaccine construct [89], and enhances immunogenicity without introducing safety concerns [90, 91].

A carefully curated set of epitopes was incorporated into the ultimate vaccine composition, exhibiting robust binding capabilities to MHC-I (*n* = 6) and MHC-II (*n *= 6) molecules alongside desirable antigenic properties. Following the identification of immunogenic epitopes and adjuvants, the construction of linker sequences is a pivotal step in developing a recombinant vaccine. To optimize the efficacy of the multi-epitope vaccine, judiciously engineered linker elements were integrated to bridge the adjuvant, cytotoxic CTL epitopes, and Helper T lymphocyte (HTL) epitopes. The (EAAAK) × 3 linker was employed to ensure structural integrity and maintain a precise spatial arrangement between the adjuvant and epitope constituents [47, 92]. To facilitate the processing and presentation of CTL epitopes, the AAY linker was utilized, enhancing the accessibility of their C-termini during antigen presentation. The GPGPG sequence was also introduced to connect the HTL epitopes, fostering an optimal HTL response [93, 94].

In silico analyses employing immunoinformatics tools comprehensively evaluated the designed multi-epitope vaccine construct’s physicochemical and structural properties. These analyses revealed favorable characteristics, including a predicted instability index of 17.16, suggesting high stability. Primary sequence analysis predicted a non-allergenic profile, a desirable feature in vaccine development. A negative GRAVY score (− 0.315) indicated a hydrophilic nature, facilitating interaction with the aqueous environment. The predicted high solubility probability (0.96) suggested enhanced solubility during overexpression in the *E. coli* system. Finally, a Vaxijen score of 0.67 indicated the potential to elicit a robust immune response, a critical aspect of an effective vaccine construct.

The molecular dynamics simulations yielded a relatively high eigenvalue of 2.5 × 10^−5^, indicating that substantial energy would be required to induce deformation at the binding interface between the recombinant vaccine construct and TLR4. This finding suggests a high degree of rigidity and structural stability in the vaccine’s conformation upon binding its target receptor, a desirable characteristic for maintaining the integrity and functionality of the vaccine-receptor complex.

Molecular dynamics simulations yielded a comparatively elevated eigenvalue (2.5 × 10^−5^), indicating that substantial energy is required to elicit deformation at the interface where the recombinant vaccine construct binds to TLR4. This finding suggests that the vaccine’s bound configuration exhibits considerable structural rigidity and stability, highly advantageous traits for maintaining the integrity and function of the vaccine–receptor complex. Such stability ensures that the vaccine remains effectively bound and can elicit the desired immune response.

Population coverage analysis is a key metric for estimating the proportion of individuals predicted to respond to a given set of epitopes with known MHC restrictions [38]. In the present study, the selected MHC class I epitopes demonstrated a high estimated population coverage of 84.72%, indicating that a large fraction of individuals worldwide are predicted to present at least one cytotoxic T lymphocyte epitope. In contrast, the MHC class II epitopes showed a lower but notable population coverage of 37.46%. Although lower than that observed for MHC class I, the combined population coverage profiles suggest that the proposed multi-epitope vaccine construct may offer meaningful coverage across diverse populations, supporting its further evaluation. Nonetheless, experimental validation remains necessary to confirm the immunogenicity and protective efficacy of the predicted epitopes across different human populations.

The coding sequence underwent a CAI analysis to evaluate the vaccine’s expression efficiency within the *E. coli* expression system. This analysis yielded a construct with a predicted solubility score of 100%, indicating exceptional potential for high-level soluble expression in the chosen host. High predicted solubility is paramount for successfully producing and purifying the recombinant multi-epitope vaccine, as it facilitates downstream processes crucial for thorough evaluation and characterization.

As SART3 is a nuclear RNA-binding protein involved in essential processes such as pre-mRNA splicing, the potential for on-target/off-tumor effects and autoimmune phenomena must be considered if normal proliferating tissues present SART3-derived epitopes. Nevertheless, early-phase clinical studies using SART3-derived peptides in patients with solid tumors have reported favorable safety profiles, with primarily mild local reactions at the injection site and no dose-limiting systemic toxicities or clear autoimmune syndromes. These findings suggest that SART3-directed vaccination can be safely administered within the tested dose ranges. In line with experience from other therapeutic cancer vaccines, the theoretical risk of autoimmunity cannot be excluded a priori. However, careful epitope selection, appropriate dosing, and rigorous preclinical and clinical monitoring are expected to mitigate on-target/off-tumor toxicity. Accordingly, our in silico construct should be regarded as a hypothesis-generating candidate requiring systematic experimental safety evaluation [8, 95, 96].

While HDOCK docking provides valuable insights into potential protein–ligand interactions, it is important to note that docking scores predict geometric fit rather than thermodynamic binding affinity or functional immune activation. Consequently, all in silico predictions—including epitope binding, structural stability, and receptor docking—require experimental confirmation.

Disulfide engineering was performed to enhance the structural rigidity and stability of the protein model by introducing covalent cysteine bridges. Among the predicted residue pairs, Val80–Ala123 exhibited the most favorable geometric parameters and the lowest bond energy, suggesting that this mutation is likely to stabilize the protein without introducing significant conformational strain. The Glu68–Thr126 pair also showed acceptable energetic values, indicating that it may serve as an alternative stabilizing mutation. In contrast, the remaining predicted pairs displayed higher bond energies, implying that their introduction could distort the local backbone conformation or interfere with proper folding. Strategic incorporation of disulfide bonds is known to reduce conformational flexibility and increase resistance to thermal or proteolytic degradation; therefore, the selected low-energy pairs may enhance the protein's structural robustness. Nevertheless, experimental validation or molecular dynamics simulations would be required to confirm whether the engineered disulfide bridges improve stability without compromising protein function.

The present results are consistent with the multi-epitope vaccine design strategy reported by Bayat et al. [42], who similarly employed immunoinformatics approaches to construct a TLR4-adjuvanted vaccine targeting the cancer/testis antigen PRAME. Both studies demonstrated favorable physicochemical properties, strong antigenicity, and predicted structural stability of the designed constructs, supporting the broader applicability of multi-epitope cancer vaccines incorporating innate immune agonists. Bayat et al. [42] utilized IEDB epitope prediction, the same *Mycobacterium tuberculosis* TLR4 agonist (L7/L12), and identical linker strategies (EAAAK, AAY, GPGPG) to assemble five MHC-I and five MHC-II epitopes into a multi-epitope construct.

In line with this, our findings are broadly consistent with the multi-epitope cancer vaccine strategy described by Baghaei et al. [41], who similarly combined predicted T-cell epitopes with a TLR4 agonist to target uPAR, another cancer-associated antigen. Both studies employed the same computational framework—IEDB epitope prediction, the *M. tuberculosis* TLR4 agonist adjuvant, five CTL and five HTL epitopes, and comprehensive in silico evaluation including molecular docking and MD simulations. Both reports also describe highly favorable vaccine characteristics: non-allergenicity, strong antigenic profiles, good solubility and physicochemical properties, and exceptionally strong predicted TLR4 binding.

The predicted docking energy of our SART3–TLR4 complex (− 265.61 kcal/mol) was slightly better than the PRAME construct (− 250.5 kcal/mol) [1] but considerably less favorable than the uPAR–TLR4 complex (− 334.37 kcal/mol) reported by Baghaei et al. [41].

Several methodological distinctions are worth noting. While Bayat et al. [1] and Baghaei et al. [41] both incorporated five MHC-I and five MHC-II epitopes, the present study utilized six epitopes from each class. Additionally, the AEAAAKEAAAKEAAAKA, AAY, and GPGPG linkers were employed for adjuvant–epitope attachment (N-terminus), MHC-I epitope joining, and MHC-II epitope linking, respectively. Immune simulation was performed in both our study and that of Baghaei et al. [41]. However, the present study executed several new analyses not included in previous reports [41, 42], including evaluation of IL-4, IL-10, and IFN-γ inducible potential, disulfide engineering, and discontinuous B-cell epitope predictions.

This computational study has several limitations. Epitope predictions, docking analyses, and physicochemical tools provide valuable screening data but do not guarantee actual immunogenicity, binding affinity, or empirical protein behavior. The vaccine construct remains theoretical and requires experimental validation of folding, epitope accessibility, and immunogenicity. Safety concerns—including on-target/off-tumor effects and TLR4 agonist reactogenicity—also require preclinical testing. Thus, this computational design serves as a hypothesis-generating foundation needing experimental confirmation.

## Conclusions

In conclusion, a novel multi-epitope vaccine targeting SART3 for cancer immunotherapy was designed and computationally evaluated. Immunoinformatics identified and integrated immunogenic T-cell epitopes. Adjuvant and linker incorporation resulted in a vaccine construct with optimal characteristics. Computational analyses predicted favorable physicochemical properties, a well-folded structure, TLR4-binding affinity, and high stability. Codon optimization suggests suitability for *E. coli* expression. These findings provide a foundation for further development, including experimental validation and preclinical studies. While limitations of in silico predictions exist, this study presents a significant step forward for SART3-targeted immunotherapy.

## Supplementary Information

Supplementary Material 1: Supplementary data 1. SART3 sequence.

Supplementary Material 2: Supplementary data 2. Experimentally validated epitopes in SART3.

Supplementary Material 3: Supplementary data 3. MHC-I extracted epitopes.

Supplementary Material 4: Supplementary data 4. MHC-II extracted epitopes.

Supplementary Material 5: Supplementary data 5. Possible multi-epitope vaccine sequences.

Supplementary Material 6: Supplementary data 6. Multi-epitope vaccine sequences evaluation.

## Data Availability

The data that support the findings of this study are available in the supplementary material of this article.
