# Supplementary material for: A bioinformatics pipeline for the design of a SART3-targeted cancer vaccine with enhanced immunogenicity
Source: Genomics Inform. 2026 May 1;24:11. doi: 10.1186/s44342-026-00068-5 (PMC13135266; doi:10.1186/s44342-026-00068-5)
Supplement: Supplementary file 1 — Supplementary Material 1: Supplementary data 1. SART3 sequence. [file 44342_2026_68_MOESM1_ESM.docx]

**SART3 in Humans and Mice (with %87 identity)**

**Human (**Q15020**); Length = 963**

>sp|Q15020|SART3_HUMAN Squamous cell carcinoma antigen recognized by T-cells 3 OS=Homo sapiens OX=9606 GN=SART3 PE=1 SV=1

MATAAETSASEPEAESKAGPKADGEEDEVKAARTRRKVLSRAVAAATYKTMGPAWDQQEE

GVSESDGDEYAMASSAESSPGEYEWEYDEEEEKNQLEIERLEEQLSINVYDYNCHVDLIR

LLRLEGELTKVRMARQKMSEIFPLTEELWLEWLHDEISMAQDGLDREHVYDLFEKAVKDY

ICPNIWLEYGQYSVGGIGQKGGLEKVRSVFERALSSVGLHMTKGLALWEAYREFESAIVE

AARLEKVHSLFRRQLAIPLYDMEATFAEYEEWSEDPIPESVIQNYNKALQQLEKYKPYEE

ALLQAEAPRLAEYQAYIDFEMKIGDPARIQLIFERALVENCLVPDLWIRYSQYLDRQLKV

KDLVLSVHNRAIRNCPWTVALWSRYLLAMERHGVDHQVISVTFEKALNAGFIQATDYVEI

WQAYLDYLRRRVDFKQDSSKELEELRAAFTRALEYLKQEVEERFNESGDPSCVIMQNWAR

IEARLCNNMQKARELWDSIMTRGNAKYANMWLEYYNLERAHGDTQHCRKALHRAVQCTSD

YPEHVCEVLLTMERTEGSLEDWDIAVQKTETRLARVNEQRMKAAEKEAALVQQEEEKAEQ

RKRARAEKKALKKKKKIRGPEKRGADEDDEKEWGDDEEEQPSKRRRVENSIPAAGETQNV

EVAAGPAGKCAAVDVEPPSKQKEKAASLKRDMPKVLHDSSKDSITVFVSNLPYSMQEPDT

KLRPLFEACGEVVQIRPIFSNRGDFRGYCYVEFKEEKSALQALEMDRKSVEGRPMFVSPC

VDKSKNPDFKVFRYSTSLEKHKLFISGLPFSCTKEELEEICKAHGTVKDLRLVTNRAGKP

KGLAYVEYENESQASQAVMKMDGMTIKENIIKVAISNPPQRKVPEKPETRKAPGGPMLLP

QTYGARGKGRTQLSLLPRALQRPSAAAPQAENGPAAAPAVAAPAATEAPKMSNADFAKLF

LRK

Length = 963

**SART3 in Mice (Q9JL18); Length = 962**

>sp|Q9JLI8|SART3_MOUSE Squamous cell carcinoma antigen recognized by T-cells 3 OS=Mus musculus OX=10090 GN=Sart3 PE=1 SV=1

MATTAASSASEPEVEPQAGPEAEGEEDEAKPAGVQRKVLSGAVAAEAAEAKGPGWDLQRE

GASGSDGDEEDAMASSAESSAGEDEWEYDEEEEKNQLEIERLEEQLSINGYDYNCHVELI

RLLRLEGELSRVRAARQKMSELFPLTEELWLEWLHDEISMAMDGLDREHVYELFERAVKD

YICPNIWLEYGQYSVGGIGQKGGLEKVRSVFERALSSVGLHMTKGLAIWEAYREFESAIV

EAARLEKVHSLFRRQLAIPLYEMEATFAEYEEWSEEPMPESVLQSYQKALGQLEKYKPYE

EALLQAEAPRLAEYQAYIDFEMKIGDPARIQLIFERALVENCLVPDLWIRYSQYLDRQLK

VKDLVLSVHSRAVRNCPWTVALWSRYLLAMERHGLDHQTISATFENALSAGFIQATDYVE

IWQVYLDYLRRRVDFRQDSSKELEELRSMFTRALEYLQQEVEERFSESGDPSCLIMQSWA

RVEARLCNNMQKARELWDSIMTRGNAKYANMWLEYYNLERAHGDTQHCRKALHRAVQCTS

DYPEHVCEVLLTMERTEGTLEDWDLAIQKTETRLARVNEQRMKAAEKEAALVQQEEEKAE

QRKKVRAEKKALKKKKKTRGADKRREDEDEENEWGEEEEEQPSKRRRTENSLASGEASAM

KEETELSGKCLTIDVGPPSKQKEKAASLKRDMPKVAHDSSKDSVTVFVSNLPYSIEEPEV

KLRPLFEVCGEVVQIRPIFSNRGDFRGYCYVEFGEEKSAQQALELDRKIVEGRPMFVSPC

VDKSKNPDFKVFRYSTTLEKHKLFISGLPFSCTKEELEDICKAHGTVKDLRLVTNRAGKP

KGLAYVEYENESQASQAVMKMDGMTIRENVIKVAISNPPQRKVPEKPEVRTAPGAPMLPR

QMYGARGKGRTQLSLLPRALQRQGAAPQAENGPAPGPAVAPSVATEAPKMSNADFAKLLL

RK
