# Supplementary material for: A bioinformatics pipeline for the design of a SART3-targeted cancer vaccine with enhanced immunogenicity
Source: Genomics Inform. 2026 May 1;24:11. doi: 10.1186/s44342-026-00068-5 (PMC13135266; doi:10.1186/s44342-026-00068-5)
Supplement: Supplementary file 5 — Supplementary Material 5: Supplementary data 5. Possible multi-epitope vaccine sequences. [file 44342_2026_68_MOESM5_ESM.docx]

**Final epitopes**

**Class-I:**

**Prediction = 3**

**Experimental = 3**

| **NPDFKVFRY** |
| --- |
| **RNCPWTVALW** |
| **YPEHVCEVL** |
| **RLEKVHSLFR** |
| **RLAEYQAYI** |
| **HVYDLFEKA** |

**Class-II:**

**Prediction = 4**

**Experimental = 2**

| **VKDLRLVTNRAGKPK** |
| --- |
| **FKVFRYSTSLEKHK** |
| **KVFRYSTSLEKHKL** |
| **EYAMASSAESSPGE** |
| **KPKGLAYVEYENES** |
| **ESVIQNYNKALQQL** |

**MAKLSTDELLDAFKEMTLLELSDFVKKFEETFEVTAAAPVAVAAAGAAPAGAAVEAAEEQSEFDVILEAAGDKKIGVIKVVREIVSGLGLKEAKDLVDGAPKPLLEKVAKEAADEAKAKLEAAGATVTVKAEAAAKEAAAKEAAAKAAAYAAYAAYAAYAAYGPGPGGPGPGGPGPGGPGPGGPGPGGPGPGGPGPGHHHHHH**

**MAKLSTDELLDAFKEMTLLELSDFVKKFEETFEVTAAAPVAVAAAGAAPAGAAVEAAEEQSEFDVILEAAGDKKIGVIKVVREIVSGLGLKEAKDLVDGAPKPLLEKVAKEAADEAKAKLEAAGATVTVKAEAAAKEAAAKEAAAKARLEKVHSLFRAAYYPEHVCEVLAAYNPDFKVFRYAAYRLAEYQAYIAAYHVYDLFEKAAAYRNCPWTVALW**

**1**

**MAKLSTDELLDAFKEMTLLELSDFVKKFEETFEVTAAAPVAVAAAGAAPAGAAVEAAEEQSEFDVILEAAGDKKIGVIKVVREIVSGLGLKEAKDLVDGAPKPLLEKVAKEAADEAKAKLEAAGATVTVKAEAAAKEAAAKEAAAKANPDFKVFRYAAYRNCPWTVALWAAYYPEHVCEVLAAYRLEKVHSLFRAAYRLAEYQAYIAAYHVYDLFEKAGPGPGVKDLRLVTNRAGKPKGPGPGFKVFRYSTSLEKHKGPGPGKVFRYSTSLEKHKLGPGPGEYAMASSAESSPGEGPGPGKPKGLAYVEYENESGPGPGESVIQNYNKALQQLGPGPGHHHHHH**

**2**

**MAKLSTDELLDAFKEMTLLELSDFVKKFEETFEVTAAAPVAVAAAGAAPAGAAVEAAEEQSEFDVILEAAGDKKIGVIKVVREIVSGLGLKEAKDLVDGAPKPLLEKVAKEAADEAKAKLEAAGATVTVKAEAAAKEAAAKEAAAKANPDFKVFRYAAYRNCPWTVALWAAYYPEHVCEVLAAYRLEKVHSLFRAAYRLAEYQAYIAAYHVYDLFEKAGPGPGVKDLRLVTNRAGKPKGPGPGKVFRYSTSLEKHKLGPGPGFKVFRYSTSLEKHKGPGPGEYAMASSAESSPGEGPGPGKPKGLAYVEYENESGPGPGESVIQNYNKALQQLGPGPGHHHHHH**

**3**

**MAKLSTDELLDAFKEMTLLELSDFVKKFEETFEVTAAAPVAVAAAGAAPAGAAVEAAEEQSEFDVILEAAGDKKIGVIKVVREIVSGLGLKEAKDLVDGAPKPLLEKVAKEAADEAKAKLEAAGATVTVKAEAAAKEAAAKEAAAKANPDFKVFRYAAYRNCPWTVALWAAYYPEHVCEVLAAYRLEKVHSLFRAAYRLAEYQAYIAAYHVYDLFEKAGPGPGVKDLRLVTNRAGKPKGPGPGKVFRYSTSLEKHKLGPGPGEYAMASSAESSPGEGPGPGFKVFRYSTSLEKHKGPGPGKPKGLAYVEYENESGPGPGESVIQNYNKALQQLGPGPGHHHHHH**

**4**

**MAKLSTDELLDAFKEMTLLELSDFVKKFEETFEVTAAAPVAVAAAGAAPAGAAVEAAEEQSEFDVILEAAGDKKIGVIKVVREIVSGLGLKEAKDLVDGAPKPLLEKVAKEAADEAKAKLEAAGATVTVKAEAAAKEAAAKEAAAKANPDFKVFRYAAYRNCPWTVALWAAYYPEHVCEVLAAYRLEKVHSLFRAAYRLAEYQAYIAAYHVYDLFEKAGPGPGVKDLRLVTNRAGKPKGPGPGKVFRYSTSLEKHKLGPGPGEYAMASSAESSPGEGPGPGKPKGLAYVEYENESGPGPGFKVFRYSTSLEKHKGPGPGESVIQNYNKALQQLGPGPGHHHHHH**

**5**

**MAKLSTDELLDAFKEMTLLELSDFVKKFEETFEVTAAAPVAVAAAGAAPAGAAVEAAEEQSEFDVILEAAGDKKIGVIKVVREIVSGLGLKEAKDLVDGAPKPLLEKVAKEAADEAKAKLEAAGATVTVKAEAAAKEAAAKEAAAKANPDFKVFRYAAYRNCPWTVALWAAYYPEHVCEVLAAYRLEKVHSLFRAAYRLAEYQAYIAAYHVYDLFEKAGPGPGVKDLRLVTNRAGKPKGPGPGKVFRYSTSLEKHKLGPGPGEYAMASSAESSPGEGPGPGKPKGLAYVEYENESGPGPGESVIQNYNKALQQLGPGPGFKVFRYSTSLEKHKGPGPGHHHHHH**

**6**

**MAKLSTDELLDAFKEMTLLELSDFVKKFEETFEVTAAAPVAVAAAGAAPAGAAVEAAEEQSEFDVILEAAGDKKIGVIKVVREIVSGLGLKEAKDLVDGAPKPLLEKVAKEAADEAKAKLEAAGATVTVKAEAAAKEAAAKEAAAKANPDFKVFRYAAYRNCPWTVALWAAYYPEHVCEVLAAYRLEKVHSLFRAAYRLAEYQAYIAAYHVYDLFEKAGPGPGFKVFRYSTSLEKHKGPGPGVKDLRLVTNRAGKPKGPGPGKVFRYSTSLEKHKLGPGPGEYAMASSAESSPGEGPGPGKPKGLAYVEYENESGPGPGESVIQNYNKALQQLGPGPGHHHHHH**

**7**

**MAKLSTDELLDAFKEMTLLELSDFVKKFEETFEVTAAAPVAVAAAGAAPAGAAVEAAEEQSEFDVILEAAGDKKIGVIKVVREIVSGLGLKEAKDLVDGAPKPLLEKVAKEAADEAKAKLEAAGATVTVKAEAAAKEAAAKEAAAKANPDFKVFRYAAYRNCPWTVALWAAYYPEHVCEVLAAYRLEKVHSLFRAAYRLAEYQAYIAAYHVYDLFEKAGPGPGFKVFRYSTSLEKHKGPGPGKVFRYSTSLEKHKLGPGPGVKDLRLVTNRAGKPKGPGPGEYAMASSAESSPGEGPGPGKPKGLAYVEYENESGPGPGESVIQNYNKALQQLGPGPGHHHHHH**

**8**

**MAKLSTDELLDAFKEMTLLELSDFVKKFEETFEVTAAAPVAVAAAGAAPAGAAVEAAEEQSEFDVILEAAGDKKIGVIKVVREIVSGLGLKEAKDLVDGAPKPLLEKVAKEAADEAKAKLEAAGATVTVKAEAAAKEAAAKEAAAKANPDFKVFRYAAYRNCPWTVALWAAYYPEHVCEVLAAYRLEKVHSLFRAAYRLAEYQAYIAAYHVYDLFEKAGPGPGFKVFRYSTSLEKHKGPGPGKVFRYSTSLEKHKLGPGPGEYAMASSAESSPGEGPGPGVKDLRLVTNRAGKPKGPGPGKPKGLAYVEYENESGPGPGESVIQNYNKALQQLGPGPGHHHHHH**

**9**

**MAKLSTDELLDAFKEMTLLELSDFVKKFEETFEVTAAAPVAVAAAGAAPAGAAVEAAEEQSEFDVILEAAGDKKIGVIKVVREIVSGLGLKEAKDLVDGAPKPLLEKVAKEAADEAKAKLEAAGATVTVKAEAAAKEAAAKEAAAKANPDFKVFRYAAYRNCPWTVALWAAYYPEHVCEVLAAYRLEKVHSLFRAAYRLAEYQAYIAAYHVYDLFEKAGPGPGFKVFRYSTSLEKHKGPGPGKVFRYSTSLEKHKLGPGPGEYAMASSAESSPGEGPGPGKPKGLAYVEYENESGPGPGVKDLRLVTNRAGKPKGPGPGESVIQNYNKALQQLGPGPGHHHHHH**

**10**

**MAKLSTDELLDAFKEMTLLELSDFVKKFEETFEVTAAAPVAVAAAGAAPAGAAVEAAEEQSEFDVILEAAGDKKIGVIKVVREIVSGLGLKEAKDLVDGAPKPLLEKVAKEAADEAKAKLEAAGATVTVKAEAAAKEAAAKEAAAKANPDFKVFRYAAYRNCPWTVALWAAYYPEHVCEVLAAYRLEKVHSLFRAAYRLAEYQAYIAAYHVYDLFEKAGPGPGFKVFRYSTSLEKHKGPGPGKVFRYSTSLEKHKLGPGPGEYAMASSAESSPGEGPGPGKPKGLAYVEYENESGPGPGESVIQNYNKALQQLGPGPGVKDLRLVTNRAGKPKGPGPGHHHHHH**

**11**

**MAKLSTDELLDAFKEMTLLELSDFVKKFEETFEVTAAAPVAVAAAGAAPAGAAVEAAEEQSEFDVILEAAGDKKIGVIKVVREIVSGLGLKEAKDLVDGAPKPLLEKVAKEAADEAKAKLEAAGATVTVKAEAAAKEAAAKEAAAKANPDFKVFRYAAYYPEHVCEVLAAYRNCPWTVALWAAYRLEKVHSLFRAAYRLAEYQAYIAAYHVYDLFEKAGPGPGVKDLRLVTNRAGKPKGPGPGFKVFRYSTSLEKHKGPGPGKVFRYSTSLEKHKLGPGPGEYAMASSAESSPGEGPGPGKPKGLAYVEYENESGPGPGESVIQNYNKALQQLGPGPGHHHHHH**

**12**

**MAKLSTDELLDAFKEMTLLELSDFVKKFEETFEVTAAAPVAVAAAGAAPAGAAVEAAEEQSEFDVILEAAGDKKIGVIKVVREIVSGLGLKEAKDLVDGAPKPLLEKVAKEAADEAKAKLEAAGATVTVKAEAAAKEAAAKEAAAKANPDFKVFRYAAYYPEHVCEVLAAYRNCPWTVALWAAYRLEKVHSLFRAAYRLAEYQAYIAAYHVYDLFEKAGPGPGVKDLRLVTNRAGKPKGPGPGKVFRYSTSLEKHKLGPGPGFKVFRYSTSLEKHKGPGPGEYAMASSAESSPGEGPGPGKPKGLAYVEYENESGPGPGESVIQNYNKALQQLGPGPGHHHHHH**

**13**

**MAKLSTDELLDAFKEMTLLELSDFVKKFEETFEVTAAAPVAVAAAGAAPAGAAVEAAEEQSEFDVILEAAGDKKIGVIKVVREIVSGLGLKEAKDLVDGAPKPLLEKVAKEAADEAKAKLEAAGATVTVKAEAAAKEAAAKEAAAKANPDFKVFRYAAYYPEHVCEVLAAYRNCPWTVALWAAYRLEKVHSLFRAAYRLAEYQAYIAAYHVYDLFEKAGPGPGVKDLRLVTNRAGKPKGPGPGKVFRYSTSLEKHKLGPGPGEYAMASSAESSPGEGPGPGFKVFRYSTSLEKHKGPGPGKPKGLAYVEYENESGPGPGESVIQNYNKALQQLGPGPGHHHHHH**

**14**

**MAKLSTDELLDAFKEMTLLELSDFVKKFEETFEVTAAAPVAVAAAGAAPAGAAVEAAEEQSEFDVILEAAGDKKIGVIKVVREIVSGLGLKEAKDLVDGAPKPLLEKVAKEAADEAKAKLEAAGATVTVKAEAAAKEAAAKEAAAKANPDFKVFRYAAYYPEHVCEVLAAYRNCPWTVALWAAYRLEKVHSLFRAAYRLAEYQAYIAAYHVYDLFEKAGPGPGVKDLRLVTNRAGKPKGPGPGKVFRYSTSLEKHKLGPGPGEYAMASSAESSPGEGPGPGKPKGLAYVEYENESGPGPGFKVFRYSTSLEKHKGPGPGESVIQNYNKALQQLGPGPGHHHHHH**

**15**

**MAKLSTDELLDAFKEMTLLELSDFVKKFEETFEVTAAAPVAVAAAGAAPAGAAVEAAEEQSEFDVILEAAGDKKIGVIKVVREIVSGLGLKEAKDLVDGAPKPLLEKVAKEAADEAKAKLEAAGATVTVKAEAAAKEAAAKEAAAKANPDFKVFRYAAYYPEHVCEVLAAYRNCPWTVALWAAYRLEKVHSLFRAAYRLAEYQAYIAAYHVYDLFEKAGPGPGVKDLRLVTNRAGKPKGPGPGKVFRYSTSLEKHKLGPGPGEYAMASSAESSPGEGPGPGKPKGLAYVEYENESGPGPGESVIQNYNKALQQLGPGPGFKVFRYSTSLEKHKGPGPGHHHHHH**

**16**

**MAKLSTDELLDAFKEMTLLELSDFVKKFEETFEVTAAAPVAVAAAGAAPAGAAVEAAEEQSEFDVILEAAGDKKIGVIKVVREIVSGLGLKEAKDLVDGAPKPLLEKVAKEAADEAKAKLEAAGATVTVKAEAAAKEAAAKEAAAKANPDFKVFRYAAYYPEHVCEVLAAYRNCPWTVALWAAYRLEKVHSLFRAAYRLAEYQAYIAAYHVYDLFEKAGPGPGFKVFRYSTSLEKHKGPGPGVKDLRLVTNRAGKPKGPGPGKVFRYSTSLEKHKLGPGPGEYAMASSAESSPGEGPGPGKPKGLAYVEYENESGPGPGESVIQNYNKALQQLGPGPGHHHHHH**

**17**

**MAKLSTDELLDAFKEMTLLELSDFVKKFEETFEVTAAAPVAVAAAGAAPAGAAVEAAEEQSEFDVILEAAGDKKIGVIKVVREIVSGLGLKEAKDLVDGAPKPLLEKVAKEAADEAKAKLEAAGATVTVKAEAAAKEAAAKEAAAKANPDFKVFRYAAYYPEHVCEVLAAYRNCPWTVALWAAYRLEKVHSLFRAAYRLAEYQAYIAAYHVYDLFEKAGPGPGFKVFRYSTSLEKHKGPGPGKVFRYSTSLEKHKLGPGPGVKDLRLVTNRAGKPKGPGPGEYAMASSAESSPGEGPGPGKPKGLAYVEYENESGPGPGESVIQNYNKALQQLGPGPGHHHHHH**

**18**

**MAKLSTDELLDAFKEMTLLELSDFVKKFEETFEVTAAAPVAVAAAGAAPAGAAVEAAEEQSEFDVILEAAGDKKIGVIKVVREIVSGLGLKEAKDLVDGAPKPLLEKVAKEAADEAKAKLEAAGATVTVKAEAAAKEAAAKEAAAKANPDFKVFRYAAYYPEHVCEVLAAYRNCPWTVALWAAYRLEKVHSLFRAAYRLAEYQAYIAAYHVYDLFEKAGPGPGFKVFRYSTSLEKHKGPGPGKVFRYSTSLEKHKLGPGPGEYAMASSAESSPGEGPGPGVKDLRLVTNRAGKPKGPGPGKPKGLAYVEYENESGPGPGESVIQNYNKALQQLGPGPGHHHHHH**

**19**

**MAKLSTDELLDAFKEMTLLELSDFVKKFEETFEVTAAAPVAVAAAGAAPAGAAVEAAEEQSEFDVILEAAGDKKIGVIKVVREIVSGLGLKEAKDLVDGAPKPLLEKVAKEAADEAKAKLEAAGATVTVKAEAAAKEAAAKEAAAKANPDFKVFRYAAYYPEHVCEVLAAYRNCPWTVALWAAYRLEKVHSLFRAAYRLAEYQAYIAAYHVYDLFEKAGPGPGFKVFRYSTSLEKHKGPGPGKVFRYSTSLEKHKLGPGPGEYAMASSAESSPGEGPGPGKPKGLAYVEYENESGPGPGVKDLRLVTNRAGKPKGPGPGESVIQNYNKALQQLGPGPGHHHHHH**

**20**

**MAKLSTDELLDAFKEMTLLELSDFVKKFEETFEVTAAAPVAVAAAGAAPAGAAVEAAEEQSEFDVILEAAGDKKIGVIKVVREIVSGLGLKEAKDLVDGAPKPLLEKVAKEAADEAKAKLEAAGATVTVKAEAAAKEAAAKEAAAKANPDFKVFRYAAYYPEHVCEVLAAYRNCPWTVALWAAYRLEKVHSLFRAAYRLAEYQAYIAAYHVYDLFEKAGPGPGFKVFRYSTSLEKHKGPGPGKVFRYSTSLEKHKLGPGPGEYAMASSAESSPGEGPGPGKPKGLAYVEYENESGPGPGESVIQNYNKALQQLGPGPGVKDLRLVTNRAGKPKGPGPGHHHHHH**

**21**

**MAKLSTDELLDAFKEMTLLELSDFVKKFEETFEVTAAAPVAVAAAGAAPAGAAVEAAEEQSEFDVILEAAGDKKIGVIKVVREIVSGLGLKEAKDLVDGAPKPLLEKVAKEAADEAKAKLEAAGATVTVKAEAAAKEAAAKEAAAKANPDFKVFRYAAYYPEHVCEVLAAYRLEKVHSLFRAAYRNCPWTVALWAAYRLAEYQAYIAAYHVYDLFEKAGPGPGVKDLRLVTNRAGKPKGPGPGFKVFRYSTSLEKHKGPGPGKVFRYSTSLEKHKLGPGPGEYAMASSAESSPGEGPGPGKPKGLAYVEYENESGPGPGESVIQNYNKALQQLGPGPGHHHHHH**

**22**

**MAKLSTDELLDAFKEMTLLELSDFVKKFEETFEVTAAAPVAVAAAGAAPAGAAVEAAEEQSEFDVILEAAGDKKIGVIKVVREIVSGLGLKEAKDLVDGAPKPLLEKVAKEAADEAKAKLEAAGATVTVKAEAAAKEAAAKEAAAKANPDFKVFRYAAYYPEHVCEVLAAYRLEKVHSLFRAAYRNCPWTVALWAAYRLAEYQAYIAAYHVYDLFEKAGPGPGVKDLRLVTNRAGKPKGPGPGKVFRYSTSLEKHKLGPGPGFKVFRYSTSLEKHKGPGPGEYAMASSAESSPGEGPGPGKPKGLAYVEYENESGPGPGESVIQNYNKALQQLGPGPGHHHHHH**

**23**

**MAKLSTDELLDAFKEMTLLELSDFVKKFEETFEVTAAAPVAVAAAGAAPAGAAVEAAEEQSEFDVILEAAGDKKIGVIKVVREIVSGLGLKEAKDLVDGAPKPLLEKVAKEAADEAKAKLEAAGATVTVKAEAAAKEAAAKEAAAKANPDFKVFRYAAYYPEHVCEVLAAYRLEKVHSLFRAAYRNCPWTVALWAAYRLAEYQAYIAAYHVYDLFEKAGPGPGVKDLRLVTNRAGKPKGPGPGKVFRYSTSLEKHKLGPGPGEYAMASSAESSPGEGPGPGFKVFRYSTSLEKHKGPGPGKPKGLAYVEYENESGPGPGESVIQNYNKALQQLGPGPGHHHHHH**

**24**

**MAKLSTDELLDAFKEMTLLELSDFVKKFEETFEVTAAAPVAVAAAGAAPAGAAVEAAEEQSEFDVILEAAGDKKIGVIKVVREIVSGLGLKEAKDLVDGAPKPLLEKVAKEAADEAKAKLEAAGATVTVKAEAAAKEAAAKEAAAKANPDFKVFRYAAYYPEHVCEVLAAYRLEKVHSLFRAAYRNCPWTVALWAAYRLAEYQAYIAAYHVYDLFEKAGPGPGVKDLRLVTNRAGKPKGPGPGKVFRYSTSLEKHKLGPGPGEYAMASSAESSPGEGPGPGKPKGLAYVEYENESGPGPGFKVFRYSTSLEKHKGPGPGESVIQNYNKALQQLGPGPGHHHHHH**

**25**

**MAKLSTDELLDAFKEMTLLELSDFVKKFEETFEVTAAAPVAVAAAGAAPAGAAVEAAEEQSEFDVILEAAGDKKIGVIKVVREIVSGLGLKEAKDLVDGAPKPLLEKVAKEAADEAKAKLEAAGATVTVKAEAAAKEAAAKEAAAKANPDFKVFRYAAYYPEHVCEVLAAYRLEKVHSLFRAAYRNCPWTVALWAAYRLAEYQAYIAAYHVYDLFEKAGPGPGVKDLRLVTNRAGKPKGPGPGKVFRYSTSLEKHKLGPGPGEYAMASSAESSPGEGPGPGKPKGLAYVEYENESGPGPGESVIQNYNKALQQLGPGPGFKVFRYSTSLEKHKGPGPGHHHHHH**

**26**

**MAKLSTDELLDAFKEMTLLELSDFVKKFEETFEVTAAAPVAVAAAGAAPAGAAVEAAEEQSEFDVILEAAGDKKIGVIKVVREIVSGLGLKEAKDLVDGAPKPLLEKVAKEAADEAKAKLEAAGATVTVKAEAAAKEAAAKEAAAKANPDFKVFRYAAYYPEHVCEVLAAYRLEKVHSLFRAAYRNCPWTVALWAAYRLAEYQAYIAAYHVYDLFEKAGPGPGFKVFRYSTSLEKHKGPGPGVKDLRLVTNRAGKPKGPGPGKVFRYSTSLEKHKLGPGPGEYAMASSAESSPGEGPGPGKPKGLAYVEYENESGPGPGESVIQNYNKALQQLGPGPGHHHHHH**

**27**

**MAKLSTDELLDAFKEMTLLELSDFVKKFEETFEVTAAAPVAVAAAGAAPAGAAVEAAEEQSEFDVILEAAGDKKIGVIKVVREIVSGLGLKEAKDLVDGAPKPLLEKVAKEAADEAKAKLEAAGATVTVKAEAAAKEAAAKEAAAKANPDFKVFRYAAYYPEHVCEVLAAYRLEKVHSLFRAAYRNCPWTVALWAAYRLAEYQAYIAAYHVYDLFEKAGPGPGFKVFRYSTSLEKHKGPGPGKVFRYSTSLEKHKLGPGPGVKDLRLVTNRAGKPKGPGPGEYAMASSAESSPGEGPGPGKPKGLAYVEYENESGPGPGESVIQNYNKALQQLGPGPGHHHHHH**

**28**

**MAKLSTDELLDAFKEMTLLELSDFVKKFEETFEVTAAAPVAVAAAGAAPAGAAVEAAEEQSEFDVILEAAGDKKIGVIKVVREIVSGLGLKEAKDLVDGAPKPLLEKVAKEAADEAKAKLEAAGATVTVKAEAAAKEAAAKEAAAKANPDFKVFRYAAYYPEHVCEVLAAYRLEKVHSLFRAAYRNCPWTVALWAAYRLAEYQAYIAAYHVYDLFEKAGPGPGFKVFRYSTSLEKHKGPGPGKVFRYSTSLEKHKLGPGPGEYAMASSAESSPGEGPGPGVKDLRLVTNRAGKPKGPGPGKPKGLAYVEYENESGPGPGESVIQNYNKALQQLGPGPGHHHHHH**

**29**

**MAKLSTDELLDAFKEMTLLELSDFVKKFEETFEVTAAAPVAVAAAGAAPAGAAVEAAEEQSEFDVILEAAGDKKIGVIKVVREIVSGLGLKEAKDLVDGAPKPLLEKVAKEAADEAKAKLEAAGATVTVKAEAAAKEAAAKEAAAKANPDFKVFRYAAYYPEHVCEVLAAYRLEKVHSLFRAAYRNCPWTVALWAAYRLAEYQAYIAAYHVYDLFEKAGPGPGFKVFRYSTSLEKHKGPGPGKVFRYSTSLEKHKLGPGPGEYAMASSAESSPGEGPGPGKPKGLAYVEYENESGPGPGVKDLRLVTNRAGKPKGPGPGESVIQNYNKALQQLGPGPGHHHHHH**

**30**

**MAKLSTDELLDAFKEMTLLELSDFVKKFEETFEVTAAAPVAVAAAGAAPAGAAVEAAEEQSEFDVILEAAGDKKIGVIKVVREIVSGLGLKEAKDLVDGAPKPLLEKVAKEAADEAKAKLEAAGATVTVKAEAAAKEAAAKEAAAKANPDFKVFRYAAYYPEHVCEVLAAYRLEKVHSLFRAAYRNCPWTVALWAAYRLAEYQAYIAAYHVYDLFEKAGPGPGFKVFRYSTSLEKHKGPGPGKVFRYSTSLEKHKLGPGPGEYAMASSAESSPGEGPGPGKPKGLAYVEYENESGPGPGESVIQNYNKALQQLGPGPGVKDLRLVTNRAGKPKGPGPGHHHHHH**

**31**

**MAKLSTDELLDAFKEMTLLELSDFVKKFEETFEVTAAAPVAVAAAGAAPAGAAVEAAEEQSEFDVILEAAGDKKIGVIKVVREIVSGLGLKEAKDLVDGAPKPLLEKVAKEAADEAKAKLEAAGATVTVKAEAAAKEAAAKEAAAKANPDFKVFRYAAYYPEHVCEVLAAYRLEKVHSLFRAAYRLAEYQAYIAAYRNCPWTVALWAAYHVYDLFEKAGPGPGVKDLRLVTNRAGKPKGPGPGFKVFRYSTSLEKHKGPGPGKVFRYSTSLEKHKLGPGPGEYAMASSAESSPGEGPGPGKPKGLAYVEYENESGPGPGESVIQNYNKALQQLGPGPGHHHHHH**

**32**

**MAKLSTDELLDAFKEMTLLELSDFVKKFEETFEVTAAAPVAVAAAGAAPAGAAVEAAEEQSEFDVILEAAGDKKIGVIKVVREIVSGLGLKEAKDLVDGAPKPLLEKVAKEAADEAKAKLEAAGATVTVKAEAAAKEAAAKEAAAKANPDFKVFRYAAYYPEHVCEVLAAYRLEKVHSLFRAAYRLAEYQAYIAAYRNCPWTVALWAAYHVYDLFEKAGPGPGVKDLRLVTNRAGKPKGPGPGKVFRYSTSLEKHKLGPGPGFKVFRYSTSLEKHKGPGPGEYAMASSAESSPGEGPGPGKPKGLAYVEYENESGPGPGESVIQNYNKALQQLGPGPGHHHHHH**

**33**

**MAKLSTDELLDAFKEMTLLELSDFVKKFEETFEVTAAAPVAVAAAGAAPAGAAVEAAEEQSEFDVILEAAGDKKIGVIKVVREIVSGLGLKEAKDLVDGAPKPLLEKVAKEAADEAKAKLEAAGATVTVKAEAAAKEAAAKEAAAKANPDFKVFRYAAYYPEHVCEVLAAYRLEKVHSLFRAAYRLAEYQAYIAAYRNCPWTVALWAAYHVYDLFEKAGPGPGVKDLRLVTNRAGKPKGPGPGKVFRYSTSLEKHKLGPGPGEYAMASSAESSPGEGPGPGFKVFRYSTSLEKHKGPGPGKPKGLAYVEYENESGPGPGESVIQNYNKALQQLGPGPGHHHHHH**

**34**

**MAKLSTDELLDAFKEMTLLELSDFVKKFEETFEVTAAAPVAVAAAGAAPAGAAVEAAEEQSEFDVILEAAGDKKIGVIKVVREIVSGLGLKEAKDLVDGAPKPLLEKVAKEAADEAKAKLEAAGATVTVKAEAAAKEAAAKEAAAKANPDFKVFRYAAYYPEHVCEVLAAYRLEKVHSLFRAAYRLAEYQAYIAAYRNCPWTVALWAAYHVYDLFEKAGPGPGVKDLRLVTNRAGKPKGPGPGKVFRYSTSLEKHKLGPGPGEYAMASSAESSPGEGPGPGKPKGLAYVEYENESGPGPGFKVFRYSTSLEKHKGPGPGESVIQNYNKALQQLGPGPGHHHHHH**

**35**

**MAKLSTDELLDAFKEMTLLELSDFVKKFEETFEVTAAAPVAVAAAGAAPAGAAVEAAEEQSEFDVILEAAGDKKIGVIKVVREIVSGLGLKEAKDLVDGAPKPLLEKVAKEAADEAKAKLEAAGATVTVKAEAAAKEAAAKEAAAKANPDFKVFRYAAYYPEHVCEVLAAYRLEKVHSLFRAAYRLAEYQAYIAAYRNCPWTVALWAAYHVYDLFEKAGPGPGVKDLRLVTNRAGKPKGPGPGKVFRYSTSLEKHKLGPGPGEYAMASSAESSPGEGPGPGKPKGLAYVEYENESGPGPGESVIQNYNKALQQLGPGPGFKVFRYSTSLEKHKGPGPGHHHHHH**

**36**

**MAKLSTDELLDAFKEMTLLELSDFVKKFEETFEVTAAAPVAVAAAGAAPAGAAVEAAEEQSEFDVILEAAGDKKIGVIKVVREIVSGLGLKEAKDLVDGAPKPLLEKVAKEAADEAKAKLEAAGATVTVKAEAAAKEAAAKEAAAKANPDFKVFRYAAYYPEHVCEVLAAYRLEKVHSLFRAAYRLAEYQAYIAAYRNCPWTVALWAAYHVYDLFEKAGPGPGFKVFRYSTSLEKHKGPGPGVKDLRLVTNRAGKPKGPGPGKVFRYSTSLEKHKLGPGPGEYAMASSAESSPGEGPGPGKPKGLAYVEYENESGPGPGESVIQNYNKALQQLGPGPGHHHHHH**

**37**

**MAKLSTDELLDAFKEMTLLELSDFVKKFEETFEVTAAAPVAVAAAGAAPAGAAVEAAEEQSEFDVILEAAGDKKIGVIKVVREIVSGLGLKEAKDLVDGAPKPLLEKVAKEAADEAKAKLEAAGATVTVKAEAAAKEAAAKEAAAKANPDFKVFRYAAYYPEHVCEVLAAYRLEKVHSLFRAAYRLAEYQAYIAAYRNCPWTVALWAAYHVYDLFEKAGPGPGFKVFRYSTSLEKHKGPGPGKVFRYSTSLEKHKLGPGPGVKDLRLVTNRAGKPKGPGPGEYAMASSAESSPGEGPGPGKPKGLAYVEYENESGPGPGESVIQNYNKALQQLGPGPGHHHHHH**

**38**

**MAKLSTDELLDAFKEMTLLELSDFVKKFEETFEVTAAAPVAVAAAGAAPAGAAVEAAEEQSEFDVILEAAGDKKIGVIKVVREIVSGLGLKEAKDLVDGAPKPLLEKVAKEAADEAKAKLEAAGATVTVKAEAAAKEAAAKEAAAKANPDFKVFRYAAYYPEHVCEVLAAYRLEKVHSLFRAAYRLAEYQAYIAAYRNCPWTVALWAAYHVYDLFEKAGPGPGFKVFRYSTSLEKHKGPGPGKVFRYSTSLEKHKLGPGPGEYAMASSAESSPGEGPGPGVKDLRLVTNRAGKPKGPGPGKPKGLAYVEYENESGPGPGESVIQNYNKALQQLGPGPGHHHHHH**

**39**

**MAKLSTDELLDAFKEMTLLELSDFVKKFEETFEVTAAAPVAVAAAGAAPAGAAVEAAEEQSEFDVILEAAGDKKIGVIKVVREIVSGLGLKEAKDLVDGAPKPLLEKVAKEAADEAKAKLEAAGATVTVKAEAAAKEAAAKEAAAKANPDFKVFRYAAYYPEHVCEVLAAYRLEKVHSLFRAAYRLAEYQAYIAAYRNCPWTVALWAAYHVYDLFEKAGPGPGFKVFRYSTSLEKHKGPGPGKVFRYSTSLEKHKLGPGPGEYAMASSAESSPGEGPGPGKPKGLAYVEYENESGPGPGVKDLRLVTNRAGKPKGPGPGESVIQNYNKALQQLGPGPGHHHHHH**

**40**

**MAKLSTDELLDAFKEMTLLELSDFVKKFEETFEVTAAAPVAVAAAGAAPAGAAVEAAEEQSEFDVILEAAGDKKIGVIKVVREIVSGLGLKEAKDLVDGAPKPLLEKVAKEAADEAKAKLEAAGATVTVKAEAAAKEAAAKEAAAKANPDFKVFRYAAYYPEHVCEVLAAYRLEKVHSLFRAAYRLAEYQAYIAAYRNCPWTVALWAAYHVYDLFEKAGPGPGFKVFRYSTSLEKHKGPGPGKVFRYSTSLEKHKLGPGPGEYAMASSAESSPGEGPGPGKPKGLAYVEYENESGPGPGESVIQNYNKALQQLGPGPGVKDLRLVTNRAGKPKGPGPGHHHHHH**

**41**

**MAKLSTDELLDAFKEMTLLELSDFVKKFEETFEVTAAAPVAVAAAGAAPAGAAVEAAEEQSEFDVILEAAGDKKIGVIKVVREIVSGLGLKEAKDLVDGAPKPLLEKVAKEAADEAKAKLEAAGATVTVKAEAAAKEAAAKEAAAKANPDFKVFRYAAYYPEHVCEVLAAYRLEKVHSLFRAAYRLAEYQAYIAAYHVYDLFEKAAAYRNCPWTVALWGPGPGVKDLRLVTNRAGKPKGPGPGFKVFRYSTSLEKHKGPGPGKVFRYSTSLEKHKLGPGPGEYAMASSAESSPGEGPGPGKPKGLAYVEYENESGPGPGESVIQNYNKALQQLGPGPGHHHHHH**

**42**

**MAKLSTDELLDAFKEMTLLELSDFVKKFEETFEVTAAAPVAVAAAGAAPAGAAVEAAEEQSEFDVILEAAGDKKIGVIKVVREIVSGLGLKEAKDLVDGAPKPLLEKVAKEAADEAKAKLEAAGATVTVKAEAAAKEAAAKEAAAKANPDFKVFRYAAYYPEHVCEVLAAYRLEKVHSLFRAAYRLAEYQAYIAAYHVYDLFEKAAAYRNCPWTVALWGPGPGVKDLRLVTNRAGKPKGPGPGKVFRYSTSLEKHKLGPGPGFKVFRYSTSLEKHKGPGPGEYAMASSAESSPGEGPGPGKPKGLAYVEYENESGPGPGESVIQNYNKALQQLGPGPGHHHHHH**

**43**

**MAKLSTDELLDAFKEMTLLELSDFVKKFEETFEVTAAAPVAVAAAGAAPAGAAVEAAEEQSEFDVILEAAGDKKIGVIKVVREIVSGLGLKEAKDLVDGAPKPLLEKVAKEAADEAKAKLEAAGATVTVKAEAAAKEAAAKEAAAKANPDFKVFRYAAYYPEHVCEVLAAYRLEKVHSLFRAAYRLAEYQAYIAAYHVYDLFEKAAAYRNCPWTVALWGPGPGVKDLRLVTNRAGKPKGPGPGKVFRYSTSLEKHKLGPGPGEYAMASSAESSPGEGPGPGFKVFRYSTSLEKHKGPGPGKPKGLAYVEYENESGPGPGESVIQNYNKALQQLGPGPGHHHHHH**

**44**

**MAKLSTDELLDAFKEMTLLELSDFVKKFEETFEVTAAAPVAVAAAGAAPAGAAVEAAEEQSEFDVILEAAGDKKIGVIKVVREIVSGLGLKEAKDLVDGAPKPLLEKVAKEAADEAKAKLEAAGATVTVKAEAAAKEAAAKEAAAKANPDFKVFRYAAYYPEHVCEVLAAYRLEKVHSLFRAAYRLAEYQAYIAAYHVYDLFEKAAAYRNCPWTVALWGPGPGVKDLRLVTNRAGKPKGPGPGKVFRYSTSLEKHKLGPGPGEYAMASSAESSPGEGPGPGKPKGLAYVEYENESGPGPGFKVFRYSTSLEKHKGPGPGESVIQNYNKALQQLGPGPGHHHHHH**

**45**

**MAKLSTDELLDAFKEMTLLELSDFVKKFEETFEVTAAAPVAVAAAGAAPAGAAVEAAEEQSEFDVILEAAGDKKIGVIKVVREIVSGLGLKEAKDLVDGAPKPLLEKVAKEAADEAKAKLEAAGATVTVKAEAAAKEAAAKEAAAKANPDFKVFRYAAYYPEHVCEVLAAYRLEKVHSLFRAAYRLAEYQAYIAAYHVYDLFEKAAAYRNCPWTVALWGPGPGVKDLRLVTNRAGKPKGPGPGKVFRYSTSLEKHKLGPGPGEYAMASSAESSPGEGPGPGKPKGLAYVEYENESGPGPGESVIQNYNKALQQLGPGPGFKVFRYSTSLEKHKGPGPGHHHHHH**

**46**

**MAKLSTDELLDAFKEMTLLELSDFVKKFEETFEVTAAAPVAVAAAGAAPAGAAVEAAEEQSEFDVILEAAGDKKIGVIKVVREIVSGLGLKEAKDLVDGAPKPLLEKVAKEAADEAKAKLEAAGATVTVKAEAAAKEAAAKEAAAKANPDFKVFRYAAYYPEHVCEVLAAYRLEKVHSLFRAAYRLAEYQAYIAAYHVYDLFEKAAAYRNCPWTVALWGPGPGFKVFRYSTSLEKHKGPGPGVKDLRLVTNRAGKPKGPGPGKVFRYSTSLEKHKLGPGPGEYAMASSAESSPGEGPGPGKPKGLAYVEYENESGPGPGESVIQNYNKALQQLGPGPGHHHHHH**

**47**

**MAKLSTDELLDAFKEMTLLELSDFVKKFEETFEVTAAAPVAVAAAGAAPAGAAVEAAEEQSEFDVILEAAGDKKIGVIKVVREIVSGLGLKEAKDLVDGAPKPLLEKVAKEAADEAKAKLEAAGATVTVKAEAAAKEAAAKEAAAKANPDFKVFRYAAYYPEHVCEVLAAYRLEKVHSLFRAAYRLAEYQAYIAAYHVYDLFEKAAAYRNCPWTVALWGPGPGFKVFRYSTSLEKHKGPGPGKVFRYSTSLEKHKLGPGPGVKDLRLVTNRAGKPKGPGPGEYAMASSAESSPGEGPGPGKPKGLAYVEYENESGPGPGESVIQNYNKALQQLGPGPGHHHHHH**

**48**

**MAKLSTDELLDAFKEMTLLELSDFVKKFEETFEVTAAAPVAVAAAGAAPAGAAVEAAEEQSEFDVILEAAGDKKIGVIKVVREIVSGLGLKEAKDLVDGAPKPLLEKVAKEAADEAKAKLEAAGATVTVKAEAAAKEAAAKEAAAKANPDFKVFRYAAYYPEHVCEVLAAYRLEKVHSLFRAAYRLAEYQAYIAAYHVYDLFEKAAAYRNCPWTVALWGPGPGFKVFRYSTSLEKHKGPGPGKVFRYSTSLEKHKLGPGPGEYAMASSAESSPGEGPGPGVKDLRLVTNRAGKPKGPGPGKPKGLAYVEYENESGPGPGESVIQNYNKALQQLGPGPGHHHHHH**

**49**

**MAKLSTDELLDAFKEMTLLELSDFVKKFEETFEVTAAAPVAVAAAGAAPAGAAVEAAEEQSEFDVILEAAGDKKIGVIKVVREIVSGLGLKEAKDLVDGAPKPLLEKVAKEAADEAKAKLEAAGATVTVKAEAAAKEAAAKEAAAKANPDFKVFRYAAYYPEHVCEVLAAYRLEKVHSLFRAAYRLAEYQAYIAAYHVYDLFEKAAAYRNCPWTVALWGPGPGFKVFRYSTSLEKHKGPGPGKVFRYSTSLEKHKLGPGPGEYAMASSAESSPGEGPGPGKPKGLAYVEYENESGPGPGVKDLRLVTNRAGKPKGPGPGESVIQNYNKALQQLGPGPGHHHHHH**

**50**

**MAKLSTDELLDAFKEMTLLELSDFVKKFEETFEVTAAAPVAVAAAGAAPAGAAVEAAEEQSEFDVILEAAGDKKIGVIKVVREIVSGLGLKEAKDLVDGAPKPLLEKVAKEAADEAKAKLEAAGATVTVKAEAAAKEAAAKEAAAKANPDFKVFRYAAYYPEHVCEVLAAYRLEKVHSLFRAAYRLAEYQAYIAAYHVYDLFEKAAAYRNCPWTVALWGPGPGFKVFRYSTSLEKHKGPGPGKVFRYSTSLEKHKLGPGPGEYAMASSAESSPGEGPGPGKPKGLAYVEYENESGPGPGESVIQNYNKALQQLGPGPGVKDLRLVTNRAGKPKGPGPGHHHHHH**

**51**

**MAKLSTDELLDAFKEMTLLELSDFVKKFEETFEVTAAAPVAVAAAGAAPAGAAVEAAEEQSEFDVILEAAGDKKIGVIKVVREIVSGLGLKEAKDLVDGAPKPLLEKVAKEAADEAKAKLEAAGATVTVKAEAAAKEAAAKEAAAKARNCPWTVALWAAYNPDFKVFRYAAYRLEKVHSLFRAAYRLAEYQAYIAAYHVYDLFEKAAAYYPEHVCEVLGPGPGVKDLRLVTNRAGKPKGPGPGFKVFRYSTSLEKHKGPGPGKVFRYSTSLEKHKLGPGPGEYAMASSAESSPGEGPGPGKPKGLAYVEYENESGPGPGESVIQNYNKALQQLGPGPGHHHHHH**

**52**

**MAKLSTDELLDAFKEMTLLELSDFVKKFEETFEVTAAAPVAVAAAGAAPAGAAVEAAEEQSEFDVILEAAGDKKIGVIKVVREIVSGLGLKEAKDLVDGAPKPLLEKVAKEAADEAKAKLEAAGATVTVKAEAAAKEAAAKEAAAKARNCPWTVALWAAYNPDFKVFRYAAYRLEKVHSLFRAAYRLAEYQAYIAAYHVYDLFEKAAAYYPEHVCEVLGPGPGVKDLRLVTNRAGKPKGPGPGKVFRYSTSLEKHKLGPGPGFKVFRYSTSLEKHKGPGPGEYAMASSAESSPGEGPGPGKPKGLAYVEYENESGPGPGESVIQNYNKALQQLGPGPGHHHHHH**

**53**

**MAKLSTDELLDAFKEMTLLELSDFVKKFEETFEVTAAAPVAVAAAGAAPAGAAVEAAEEQSEFDVILEAAGDKKIGVIKVVREIVSGLGLKEAKDLVDGAPKPLLEKVAKEAADEAKAKLEAAGATVTVKAEAAAKEAAAKEAAAKARNCPWTVALWAAYNPDFKVFRYAAYRLEKVHSLFRAAYRLAEYQAYIAAYHVYDLFEKAAAYYPEHVCEVLGPGPGVKDLRLVTNRAGKPKGPGPGKVFRYSTSLEKHKLGPGPGEYAMASSAESSPGEGPGPGFKVFRYSTSLEKHKGPGPGKPKGLAYVEYENESGPGPGESVIQNYNKALQQLGPGPGHHHHHH**

**54**

**MAKLSTDELLDAFKEMTLLELSDFVKKFEETFEVTAAAPVAVAAAGAAPAGAAVEAAEEQSEFDVILEAAGDKKIGVIKVVREIVSGLGLKEAKDLVDGAPKPLLEKVAKEAADEAKAKLEAAGATVTVKAEAAAKEAAAKEAAAKARNCPWTVALWAAYNPDFKVFRYAAYRLEKVHSLFRAAYRLAEYQAYIAAYHVYDLFEKAAAYYPEHVCEVLGPGPGVKDLRLVTNRAGKPKGPGPGKVFRYSTSLEKHKLGPGPGEYAMASSAESSPGEGPGPGKPKGLAYVEYENESGPGPGFKVFRYSTSLEKHKGPGPGESVIQNYNKALQQLGPGPGHHHHHH**

**55**

**MAKLSTDELLDAFKEMTLLELSDFVKKFEETFEVTAAAPVAVAAAGAAPAGAAVEAAEEQSEFDVILEAAGDKKIGVIKVVREIVSGLGLKEAKDLVDGAPKPLLEKVAKEAADEAKAKLEAAGATVTVKAEAAAKEAAAKEAAAKARNCPWTVALWAAYNPDFKVFRYAAYRLEKVHSLFRAAYRLAEYQAYIAAYHVYDLFEKAAAYYPEHVCEVLGPGPGVKDLRLVTNRAGKPKGPGPGKVFRYSTSLEKHKLGPGPGEYAMASSAESSPGEGPGPGKPKGLAYVEYENESGPGPGESVIQNYNKALQQLGPGPGFKVFRYSTSLEKHKGPGPGHHHHHH**

**56**

**MAKLSTDELLDAFKEMTLLELSDFVKKFEETFEVTAAAPVAVAAAGAAPAGAAVEAAEEQSEFDVILEAAGDKKIGVIKVVREIVSGLGLKEAKDLVDGAPKPLLEKVAKEAADEAKAKLEAAGATVTVKAEAAAKEAAAKEAAAKARNCPWTVALWAAYNPDFKVFRYAAYRLEKVHSLFRAAYRLAEYQAYIAAYHVYDLFEKAAAYYPEHVCEVLGPGPGFKVFRYSTSLEKHKGPGPGVKDLRLVTNRAGKPKGPGPGKVFRYSTSLEKHKLGPGPGEYAMASSAESSPGEGPGPGKPKGLAYVEYENESGPGPGESVIQNYNKALQQLGPGPGHHHHHH**

**57**

**MAKLSTDELLDAFKEMTLLELSDFVKKFEETFEVTAAAPVAVAAAGAAPAGAAVEAAEEQSEFDVILEAAGDKKIGVIKVVREIVSGLGLKEAKDLVDGAPKPLLEKVAKEAADEAKAKLEAAGATVTVKAEAAAKEAAAKEAAAKARNCPWTVALWAAYNPDFKVFRYAAYRLEKVHSLFRAAYRLAEYQAYIAAYHVYDLFEKAAAYYPEHVCEVLGPGPGFKVFRYSTSLEKHKGPGPGKVFRYSTSLEKHKLGPGPGVKDLRLVTNRAGKPKGPGPGEYAMASSAESSPGEGPGPGKPKGLAYVEYENESGPGPGESVIQNYNKALQQLGPGPGHHHHHH**

**58**

**MAKLSTDELLDAFKEMTLLELSDFVKKFEETFEVTAAAPVAVAAAGAAPAGAAVEAAEEQSEFDVILEAAGDKKIGVIKVVREIVSGLGLKEAKDLVDGAPKPLLEKVAKEAADEAKAKLEAAGATVTVKAEAAAKEAAAKEAAAKARNCPWTVALWAAYNPDFKVFRYAAYRLEKVHSLFRAAYRLAEYQAYIAAYHVYDLFEKAAAYYPEHVCEVLGPGPGFKVFRYSTSLEKHKGPGPGKVFRYSTSLEKHKLGPGPGEYAMASSAESSPGEGPGPGVKDLRLVTNRAGKPKGPGPGKPKGLAYVEYENESGPGPGESVIQNYNKALQQLGPGPGHHHHHH**

**59**

**MAKLSTDELLDAFKEMTLLELSDFVKKFEETFEVTAAAPVAVAAAGAAPAGAAVEAAEEQSEFDVILEAAGDKKIGVIKVVREIVSGLGLKEAKDLVDGAPKPLLEKVAKEAADEAKAKLEAAGATVTVKAEAAAKEAAAKEAAAKARNCPWTVALWAAYNPDFKVFRYAAYRLEKVHSLFRAAYRLAEYQAYIAAYHVYDLFEKAAAYYPEHVCEVLGPGPGFKVFRYSTSLEKHKGPGPGKVFRYSTSLEKHKLGPGPGEYAMASSAESSPGEGPGPGKPKGLAYVEYENESGPGPGVKDLRLVTNRAGKPKGPGPGESVIQNYNKALQQLGPGPGHHHHHH**

**60**

**MAKLSTDELLDAFKEMTLLELSDFVKKFEETFEVTAAAPVAVAAAGAAPAGAAVEAAEEQSEFDVILEAAGDKKIGVIKVVREIVSGLGLKEAKDLVDGAPKPLLEKVAKEAADEAKAKLEAAGATVTVKAEAAAKEAAAKEAAAKARNCPWTVALWAAYNPDFKVFRYAAYRLEKVHSLFRAAYRLAEYQAYIAAYHVYDLFEKAAAYYPEHVCEVLGPGPGFKVFRYSTSLEKHKGPGPGKVFRYSTSLEKHKLGPGPGEYAMASSAESSPGEGPGPGKPKGLAYVEYENESGPGPGESVIQNYNKALQQLGPGPGVKDLRLVTNRAGKPKGPGPGHHHHHH**

**61**

**MAKLSTDELLDAFKEMTLLELSDFVKKFEETFEVTAAAPVAVAAAGAAPAGAAVEAAEEQSEFDVILEAAGDKKIGVIKVVREIVSGLGLKEAKDLVDGAPKPLLEKVAKEAADEAKAKLEAAGATVTVKAEAAAKEAAAKEAAAKARNCPWTVALWAAYNPDFKVFRYAAYRLEKVHSLFRAAYYPEHVCEVLAAYHVYDLFEKAAAYRLAEYQAYIGPGPGVKDLRLVTNRAGKPKGPGPGFKVFRYSTSLEKHKGPGPGKVFRYSTSLEKHKLGPGPGEYAMASSAESSPGEGPGPGKPKGLAYVEYENESGPGPGESVIQNYNKALQQLGPGPGHHHHHH**

**62**

**MAKLSTDELLDAFKEMTLLELSDFVKKFEETFEVTAAAPVAVAAAGAAPAGAAVEAAEEQSEFDVILEAAGDKKIGVIKVVREIVSGLGLKEAKDLVDGAPKPLLEKVAKEAADEAKAKLEAAGATVTVKAEAAAKEAAAKEAAAKARNCPWTVALWAAYNPDFKVFRYAAYRLEKVHSLFRAAYYPEHVCEVLAAYHVYDLFEKAAAYRLAEYQAYIGPGPGVKDLRLVTNRAGKPKGPGPGKVFRYSTSLEKHKLGPGPGFKVFRYSTSLEKHKGPGPGEYAMASSAESSPGEGPGPGKPKGLAYVEYENESGPGPGESVIQNYNKALQQLGPGPGHHHHHH**

**63**

**MAKLSTDELLDAFKEMTLLELSDFVKKFEETFEVTAAAPVAVAAAGAAPAGAAVEAAEEQSEFDVILEAAGDKKIGVIKVVREIVSGLGLKEAKDLVDGAPKPLLEKVAKEAADEAKAKLEAAGATVTVKAEAAAKEAAAKEAAAKARNCPWTVALWAAYNPDFKVFRYAAYRLEKVHSLFRAAYYPEHVCEVLAAYHVYDLFEKAAAYRLAEYQAYIGPGPGVKDLRLVTNRAGKPKGPGPGKVFRYSTSLEKHKLGPGPGEYAMASSAESSPGEGPGPGFKVFRYSTSLEKHKGPGPGKPKGLAYVEYENESGPGPGESVIQNYNKALQQLGPGPGHHHHHH**

**64**

**MAKLSTDELLDAFKEMTLLELSDFVKKFEETFEVTAAAPVAVAAAGAAPAGAAVEAAEEQSEFDVILEAAGDKKIGVIKVVREIVSGLGLKEAKDLVDGAPKPLLEKVAKEAADEAKAKLEAAGATVTVKAEAAAKEAAAKEAAAKARNCPWTVALWAAYNPDFKVFRYAAYRLEKVHSLFRAAYYPEHVCEVLAAYHVYDLFEKAAAYRLAEYQAYIGPGPGVKDLRLVTNRAGKPKGPGPGKVFRYSTSLEKHKLGPGPGEYAMASSAESSPGEGPGPGKPKGLAYVEYENESGPGPGFKVFRYSTSLEKHKGPGPGESVIQNYNKALQQLGPGPGHHHHHH**

**65**

**MAKLSTDELLDAFKEMTLLELSDFVKKFEETFEVTAAAPVAVAAAGAAPAGAAVEAAEEQSEFDVILEAAGDKKIGVIKVVREIVSGLGLKEAKDLVDGAPKPLLEKVAKEAADEAKAKLEAAGATVTVKAEAAAKEAAAKEAAAKARNCPWTVALWAAYNPDFKVFRYAAYRLEKVHSLFRAAYYPEHVCEVLAAYHVYDLFEKAAAYRLAEYQAYIGPGPGVKDLRLVTNRAGKPKGPGPGKVFRYSTSLEKHKLGPGPGEYAMASSAESSPGEGPGPGKPKGLAYVEYENESGPGPGESVIQNYNKALQQLGPGPGFKVFRYSTSLEKHKGPGPGHHHHHH**

**66**

**MAKLSTDELLDAFKEMTLLELSDFVKKFEETFEVTAAAPVAVAAAGAAPAGAAVEAAEEQSEFDVILEAAGDKKIGVIKVVREIVSGLGLKEAKDLVDGAPKPLLEKVAKEAADEAKAKLEAAGATVTVKAEAAAKEAAAKEAAAKARNCPWTVALWAAYNPDFKVFRYAAYRLEKVHSLFRAAYYPEHVCEVLAAYHVYDLFEKAAAYRLAEYQAYIGPGPGFKVFRYSTSLEKHKGPGPGVKDLRLVTNRAGKPKGPGPGKVFRYSTSLEKHKLGPGPGEYAMASSAESSPGEGPGPGKPKGLAYVEYENESGPGPGESVIQNYNKALQQLGPGPGHHHHHH**

**67**

**MAKLSTDELLDAFKEMTLLELSDFVKKFEETFEVTAAAPVAVAAAGAAPAGAAVEAAEEQSEFDVILEAAGDKKIGVIKVVREIVSGLGLKEAKDLVDGAPKPLLEKVAKEAADEAKAKLEAAGATVTVKAEAAAKEAAAKEAAAKARNCPWTVALWAAYNPDFKVFRYAAYRLEKVHSLFRAAYYPEHVCEVLAAYHVYDLFEKAAAYRLAEYQAYIGPGPGFKVFRYSTSLEKHKGPGPGKVFRYSTSLEKHKLGPGPGVKDLRLVTNRAGKPKGPGPGEYAMASSAESSPGEGPGPGKPKGLAYVEYENESGPGPGESVIQNYNKALQQLGPGPGHHHHHH**

**68**

**MAKLSTDELLDAFKEMTLLELSDFVKKFEETFEVTAAAPVAVAAAGAAPAGAAVEAAEEQSEFDVILEAAGDKKIGVIKVVREIVSGLGLKEAKDLVDGAPKPLLEKVAKEAADEAKAKLEAAGATVTVKAEAAAKEAAAKEAAAKARNCPWTVALWAAYNPDFKVFRYAAYRLEKVHSLFRAAYYPEHVCEVLAAYHVYDLFEKAAAYRLAEYQAYIGPGPGFKVFRYSTSLEKHKGPGPGKVFRYSTSLEKHKLGPGPGEYAMASSAESSPGEGPGPGVKDLRLVTNRAGKPKGPGPGKPKGLAYVEYENESGPGPGESVIQNYNKALQQLGPGPGHHHHHH**

**69**

**MAKLSTDELLDAFKEMTLLELSDFVKKFEETFEVTAAAPVAVAAAGAAPAGAAVEAAEEQSEFDVILEAAGDKKIGVIKVVREIVSGLGLKEAKDLVDGAPKPLLEKVAKEAADEAKAKLEAAGATVTVKAEAAAKEAAAKEAAAKARNCPWTVALWAAYNPDFKVFRYAAYRLEKVHSLFRAAYYPEHVCEVLAAYHVYDLFEKAAAYRLAEYQAYIGPGPGFKVFRYSTSLEKHKGPGPGKVFRYSTSLEKHKLGPGPGEYAMASSAESSPGEGPGPGKPKGLAYVEYENESGPGPGVKDLRLVTNRAGKPKGPGPGESVIQNYNKALQQLGPGPGHHHHHH**

**70**

**MAKLSTDELLDAFKEMTLLELSDFVKKFEETFEVTAAAPVAVAAAGAAPAGAAVEAAEEQSEFDVILEAAGDKKIGVIKVVREIVSGLGLKEAKDLVDGAPKPLLEKVAKEAADEAKAKLEAAGATVTVKAEAAAKEAAAKEAAAKARNCPWTVALWAAYNPDFKVFRYAAYRLEKVHSLFRAAYYPEHVCEVLAAYHVYDLFEKAAAYRLAEYQAYIGPGPGFKVFRYSTSLEKHKGPGPGKVFRYSTSLEKHKLGPGPGEYAMASSAESSPGEGPGPGKPKGLAYVEYENESGPGPGESVIQNYNKALQQLGPGPGVKDLRLVTNRAGKPKGPGPGHHHHHH**

**71**

**MAKLSTDELLDAFKEMTLLELSDFVKKFEETFEVTAAAPVAVAAAGAAPAGAAVEAAEEQSEFDVILEAAGDKKIGVIKVVREIVSGLGLKEAKDLVDGAPKPLLEKVAKEAADEAKAKLEAAGATVTVKAEAAAKEAAAKEAAAKARNCPWTVALWAAYYPEHVCEVLAAYRLEKVHSLFRAAYRLAEYQAYIAAYHVYDLFEKAAAYNPDFKVFRYGPGPGVKDLRLVTNRAGKPKGPGPGFKVFRYSTSLEKHKGPGPGKVFRYSTSLEKHKLGPGPGEYAMASSAESSPGEGPGPGKPKGLAYVEYENESGPGPGESVIQNYNKALQQLGPGPGHHHHHH**

**72**

**MAKLSTDELLDAFKEMTLLELSDFVKKFEETFEVTAAAPVAVAAAGAAPAGAAVEAAEEQSEFDVILEAAGDKKIGVIKVVREIVSGLGLKEAKDLVDGAPKPLLEKVAKEAADEAKAKLEAAGATVTVKAEAAAKEAAAKEAAAKARNCPWTVALWAAYYPEHVCEVLAAYRLEKVHSLFRAAYRLAEYQAYIAAYHVYDLFEKAAAYNPDFKVFRYGPGPGVKDLRLVTNRAGKPKGPGPGKVFRYSTSLEKHKLGPGPGFKVFRYSTSLEKHKGPGPGEYAMASSAESSPGEGPGPGKPKGLAYVEYENESGPGPGESVIQNYNKALQQLGPGPGHHHHHH**

**73**

**MAKLSTDELLDAFKEMTLLELSDFVKKFEETFEVTAAAPVAVAAAGAAPAGAAVEAAEEQSEFDVILEAAGDKKIGVIKVVREIVSGLGLKEAKDLVDGAPKPLLEKVAKEAADEAKAKLEAAGATVTVKAEAAAKEAAAKEAAAKARNCPWTVALWAAYYPEHVCEVLAAYRLEKVHSLFRAAYRLAEYQAYIAAYHVYDLFEKAAAYNPDFKVFRYGPGPGVKDLRLVTNRAGKPKGPGPGKVFRYSTSLEKHKLGPGPGEYAMASSAESSPGEGPGPGFKVFRYSTSLEKHKGPGPGKPKGLAYVEYENESGPGPGESVIQNYNKALQQLGPGPGHHHHHH**

**74**

**MAKLSTDELLDAFKEMTLLELSDFVKKFEETFEVTAAAPVAVAAAGAAPAGAAVEAAEEQSEFDVILEAAGDKKIGVIKVVREIVSGLGLKEAKDLVDGAPKPLLEKVAKEAADEAKAKLEAAGATVTVKAEAAAKEAAAKEAAAKARNCPWTVALWAAYYPEHVCEVLAAYRLEKVHSLFRAAYRLAEYQAYIAAYHVYDLFEKAAAYNPDFKVFRYGPGPGVKDLRLVTNRAGKPKGPGPGKVFRYSTSLEKHKLGPGPGEYAMASSAESSPGEGPGPGKPKGLAYVEYENESGPGPGFKVFRYSTSLEKHKGPGPGESVIQNYNKALQQLGPGPGHHHHHH**

**75**

**MAKLSTDELLDAFKEMTLLELSDFVKKFEETFEVTAAAPVAVAAAGAAPAGAAVEAAEEQSEFDVILEAAGDKKIGVIKVVREIVSGLGLKEAKDLVDGAPKPLLEKVAKEAADEAKAKLEAAGATVTVKAEAAAKEAAAKEAAAKARNCPWTVALWAAYYPEHVCEVLAAYRLEKVHSLFRAAYRLAEYQAYIAAYHVYDLFEKAAAYNPDFKVFRYGPGPGVKDLRLVTNRAGKPKGPGPGKVFRYSTSLEKHKLGPGPGEYAMASSAESSPGEGPGPGKPKGLAYVEYENESGPGPGESVIQNYNKALQQLGPGPGFKVFRYSTSLEKHKGPGPGHHHHHH**

**76**

**MAKLSTDELLDAFKEMTLLELSDFVKKFEETFEVTAAAPVAVAAAGAAPAGAAVEAAEEQSEFDVILEAAGDKKIGVIKVVREIVSGLGLKEAKDLVDGAPKPLLEKVAKEAADEAKAKLEAAGATVTVKAEAAAKEAAAKEAAAKARNCPWTVALWAAYYPEHVCEVLAAYRLEKVHSLFRAAYRLAEYQAYIAAYHVYDLFEKAAAYNPDFKVFRYGPGPGFKVFRYSTSLEKHKGPGPGVKDLRLVTNRAGKPKGPGPGKVFRYSTSLEKHKLGPGPGEYAMASSAESSPGEGPGPGKPKGLAYVEYENESGPGPGESVIQNYNKALQQLGPGPGHHHHHH**

**77**

**MAKLSTDELLDAFKEMTLLELSDFVKKFEETFEVTAAAPVAVAAAGAAPAGAAVEAAEEQSEFDVILEAAGDKKIGVIKVVREIVSGLGLKEAKDLVDGAPKPLLEKVAKEAADEAKAKLEAAGATVTVKAEAAAKEAAAKEAAAKARNCPWTVALWAAYYPEHVCEVLAAYRLEKVHSLFRAAYRLAEYQAYIAAYHVYDLFEKAAAYNPDFKVFRYGPGPGFKVFRYSTSLEKHKGPGPGKVFRYSTSLEKHKLGPGPGVKDLRLVTNRAGKPKGPGPGEYAMASSAESSPGEGPGPGKPKGLAYVEYENESGPGPGESVIQNYNKALQQLGPGPGHHHHHH**

**78**

**MAKLSTDELLDAFKEMTLLELSDFVKKFEETFEVTAAAPVAVAAAGAAPAGAAVEAAEEQSEFDVILEAAGDKKIGVIKVVREIVSGLGLKEAKDLVDGAPKPLLEKVAKEAADEAKAKLEAAGATVTVKAEAAAKEAAAKEAAAKARNCPWTVALWAAYYPEHVCEVLAAYRLEKVHSLFRAAYRLAEYQAYIAAYHVYDLFEKAAAYNPDFKVFRYGPGPGFKVFRYSTSLEKHKGPGPGKVFRYSTSLEKHKLGPGPGEYAMASSAESSPGEGPGPGVKDLRLVTNRAGKPKGPGPGKPKGLAYVEYENESGPGPGESVIQNYNKALQQLGPGPGHHHHHH**

**79**

**MAKLSTDELLDAFKEMTLLELSDFVKKFEETFEVTAAAPVAVAAAGAAPAGAAVEAAEEQSEFDVILEAAGDKKIGVIKVVREIVSGLGLKEAKDLVDGAPKPLLEKVAKEAADEAKAKLEAAGATVTVKAEAAAKEAAAKEAAAKARNCPWTVALWAAYYPEHVCEVLAAYRLEKVHSLFRAAYRLAEYQAYIAAYHVYDLFEKAAAYNPDFKVFRYGPGPGFKVFRYSTSLEKHKGPGPGKVFRYSTSLEKHKLGPGPGEYAMASSAESSPGEGPGPGKPKGLAYVEYENESGPGPGVKDLRLVTNRAGKPKGPGPGESVIQNYNKALQQLGPGPGHHHHHH**

**80**

**MAKLSTDELLDAFKEMTLLELSDFVKKFEETFEVTAAAPVAVAAAGAAPAGAAVEAAEEQSEFDVILEAAGDKKIGVIKVVREIVSGLGLKEAKDLVDGAPKPLLEKVAKEAADEAKAKLEAAGATVTVKAEAAAKEAAAKEAAAKARNCPWTVALWAAYYPEHVCEVLAAYRLEKVHSLFRAAYRLAEYQAYIAAYHVYDLFEKAAAYNPDFKVFRYGPGPGFKVFRYSTSLEKHKGPGPGKVFRYSTSLEKHKLGPGPGEYAMASSAESSPGEGPGPGKPKGLAYVEYENESGPGPGESVIQNYNKALQQLGPGPGVKDLRLVTNRAGKPKGPGPGHHHHHH**

**81**

**MAKLSTDELLDAFKEMTLLELSDFVKKFEETFEVTAAAPVAVAAAGAAPAGAAVEAAEEQSEFDVILEAAGDKKIGVIKVVREIVSGLGLKEAKDLVDGAPKPLLEKVAKEAADEAKAKLEAAGATVTVKAEAAAKEAAAKEAAAKARNCPWTVALWAAYYPEHVCEVLAAYNPDFKVFRYAAYRLAEYQAYIAAYHVYDLFEKAAAYRLEKVHSLFRGPGPGVKDLRLVTNRAGKPKGPGPGFKVFRYSTSLEKHKGPGPGKVFRYSTSLEKHKLGPGPGEYAMASSAESSPGEGPGPGKPKGLAYVEYENESGPGPGESVIQNYNKALQQLGPGPGHHHHHH**

**82**

**MAKLSTDELLDAFKEMTLLELSDFVKKFEETFEVTAAAPVAVAAAGAAPAGAAVEAAEEQSEFDVILEAAGDKKIGVIKVVREIVSGLGLKEAKDLVDGAPKPLLEKVAKEAADEAKAKLEAAGATVTVKAEAAAKEAAAKEAAAKARNCPWTVALWAAYYPEHVCEVLAAYNPDFKVFRYAAYRLAEYQAYIAAYHVYDLFEKAAAYRLEKVHSLFRGPGPGVKDLRLVTNRAGKPKGPGPGKVFRYSTSLEKHKLGPGPGFKVFRYSTSLEKHKGPGPGEYAMASSAESSPGEGPGPGKPKGLAYVEYENESGPGPGESVIQNYNKALQQLGPGPGHHHHHH**

**83**

**MAKLSTDELLDAFKEMTLLELSDFVKKFEETFEVTAAAPVAVAAAGAAPAGAAVEAAEEQSEFDVILEAAGDKKIGVIKVVREIVSGLGLKEAKDLVDGAPKPLLEKVAKEAADEAKAKLEAAGATVTVKAEAAAKEAAAKEAAAKARNCPWTVALWAAYYPEHVCEVLAAYNPDFKVFRYAAYRLAEYQAYIAAYHVYDLFEKAAAYRLEKVHSLFRGPGPGVKDLRLVTNRAGKPKGPGPGKVFRYSTSLEKHKLGPGPGEYAMASSAESSPGEGPGPGFKVFRYSTSLEKHKGPGPGKPKGLAYVEYENESGPGPGESVIQNYNKALQQLGPGPGHHHHHH**

**84**

**MAKLSTDELLDAFKEMTLLELSDFVKKFEETFEVTAAAPVAVAAAGAAPAGAAVEAAEEQSEFDVILEAAGDKKIGVIKVVREIVSGLGLKEAKDLVDGAPKPLLEKVAKEAADEAKAKLEAAGATVTVKAEAAAKEAAAKEAAAKARNCPWTVALWAAYYPEHVCEVLAAYNPDFKVFRYAAYRLAEYQAYIAAYHVYDLFEKAAAYRLEKVHSLFRGPGPGVKDLRLVTNRAGKPKGPGPGKVFRYSTSLEKHKLGPGPGEYAMASSAESSPGEGPGPGKPKGLAYVEYENESGPGPGFKVFRYSTSLEKHKGPGPGESVIQNYNKALQQLGPGPGHHHHHH**

**85**

**MAKLSTDELLDAFKEMTLLELSDFVKKFEETFEVTAAAPVAVAAAGAAPAGAAVEAAEEQSEFDVILEAAGDKKIGVIKVVREIVSGLGLKEAKDLVDGAPKPLLEKVAKEAADEAKAKLEAAGATVTVKAEAAAKEAAAKEAAAKARNCPWTVALWAAYYPEHVCEVLAAYNPDFKVFRYAAYRLAEYQAYIAAYHVYDLFEKAAAYRLEKVHSLFRGPGPGVKDLRLVTNRAGKPKGPGPGKVFRYSTSLEKHKLGPGPGEYAMASSAESSPGEGPGPGKPKGLAYVEYENESGPGPGESVIQNYNKALQQLGPGPGFKVFRYSTSLEKHKGPGPGHHHHHH**

**86**

**MAKLSTDELLDAFKEMTLLELSDFVKKFEETFEVTAAAPVAVAAAGAAPAGAAVEAAEEQSEFDVILEAAGDKKIGVIKVVREIVSGLGLKEAKDLVDGAPKPLLEKVAKEAADEAKAKLEAAGATVTVKAEAAAKEAAAKEAAAKARNCPWTVALWAAYYPEHVCEVLAAYNPDFKVFRYAAYRLAEYQAYIAAYHVYDLFEKAAAYRLEKVHSLFRGPGPGFKVFRYSTSLEKHKGPGPGVKDLRLVTNRAGKPKGPGPGKVFRYSTSLEKHKLGPGPGEYAMASSAESSPGEGPGPGKPKGLAYVEYENESGPGPGESVIQNYNKALQQLGPGPGHHHHHH**

**87**

**MAKLSTDELLDAFKEMTLLELSDFVKKFEETFEVTAAAPVAVAAAGAAPAGAAVEAAEEQSEFDVILEAAGDKKIGVIKVVREIVSGLGLKEAKDLVDGAPKPLLEKVAKEAADEAKAKLEAAGATVTVKAEAAAKEAAAKEAAAKARNCPWTVALWAAYYPEHVCEVLAAYNPDFKVFRYAAYRLAEYQAYIAAYHVYDLFEKAAAYRLEKVHSLFRGPGPGFKVFRYSTSLEKHKGPGPGKVFRYSTSLEKHKLGPGPGVKDLRLVTNRAGKPKGPGPGEYAMASSAESSPGEGPGPGKPKGLAYVEYENESGPGPGESVIQNYNKALQQLGPGPGHHHHHH**

**88**

**MAKLSTDELLDAFKEMTLLELSDFVKKFEETFEVTAAAPVAVAAAGAAPAGAAVEAAEEQSEFDVILEAAGDKKIGVIKVVREIVSGLGLKEAKDLVDGAPKPLLEKVAKEAADEAKAKLEAAGATVTVKAEAAAKEAAAKEAAAKARNCPWTVALWAAYYPEHVCEVLAAYNPDFKVFRYAAYRLAEYQAYIAAYHVYDLFEKAAAYRLEKVHSLFRGPGPGFKVFRYSTSLEKHKGPGPGKVFRYSTSLEKHKLGPGPGEYAMASSAESSPGEGPGPGVKDLRLVTNRAGKPKGPGPGKPKGLAYVEYENESGPGPGESVIQNYNKALQQLGPGPGHHHHHH**

**89**

**MAKLSTDELLDAFKEMTLLELSDFVKKFEETFEVTAAAPVAVAAAGAAPAGAAVEAAEEQSEFDVILEAAGDKKIGVIKVVREIVSGLGLKEAKDLVDGAPKPLLEKVAKEAADEAKAKLEAAGATVTVKAEAAAKEAAAKEAAAKARNCPWTVALWAAYYPEHVCEVLAAYNPDFKVFRYAAYRLAEYQAYIAAYHVYDLFEKAAAYRLEKVHSLFRGPGPGFKVFRYSTSLEKHKGPGPGKVFRYSTSLEKHKLGPGPGEYAMASSAESSPGEGPGPGKPKGLAYVEYENESGPGPGVKDLRLVTNRAGKPKGPGPGESVIQNYNKALQQLGPGPGHHHHHH**

**90**

**MAKLSTDELLDAFKEMTLLELSDFVKKFEETFEVTAAAPVAVAAAGAAPAGAAVEAAEEQSEFDVILEAAGDKKIGVIKVVREIVSGLGLKEAKDLVDGAPKPLLEKVAKEAADEAKAKLEAAGATVTVKAEAAAKEAAAKEAAAKARNCPWTVALWAAYYPEHVCEVLAAYNPDFKVFRYAAYRLAEYQAYIAAYHVYDLFEKAAAYRLEKVHSLFRGPGPGFKVFRYSTSLEKHKGPGPGKVFRYSTSLEKHKLGPGPGEYAMASSAESSPGEGPGPGKPKGLAYVEYENESGPGPGESVIQNYNKALQQLGPGPGVKDLRLVTNRAGKPKGPGPGHHHHHH**

**91**

**MAKLSTDELLDAFKEMTLLELSDFVKKFEETFEVTAAAPVAVAAAGAAPAGAAVEAAEEQSEFDVILEAAGDKKIGVIKVVREIVSGLGLKEAKDLVDGAPKPLLEKVAKEAADEAKAKLEAAGATVTVKAEAAAKEAAAKEAAAKAYPEHVCEVLAAYRNCPWTVALWAAYNPDFKVFRYAAYRLAEYQAYIAAYHVYDLFEKAAAYRLEKVHSLFRGPGPGVKDLRLVTNRAGKPKGPGPGFKVFRYSTSLEKHKGPGPGKVFRYSTSLEKHKLGPGPGEYAMASSAESSPGEGPGPGKPKGLAYVEYENESGPGPGESVIQNYNKALQQLGPGPGHHHHHH**

**92**

**MAKLSTDELLDAFKEMTLLELSDFVKKFEETFEVTAAAPVAVAAAGAAPAGAAVEAAEEQSEFDVILEAAGDKKIGVIKVVREIVSGLGLKEAKDLVDGAPKPLLEKVAKEAADEAKAKLEAAGATVTVKAEAAAKEAAAKEAAAKAYPEHVCEVLAAYRNCPWTVALWAAYNPDFKVFRYAAYRLAEYQAYIAAYHVYDLFEKAAAYRLEKVHSLFRGPGPGVKDLRLVTNRAGKPKGPGPGKVFRYSTSLEKHKLGPGPGFKVFRYSTSLEKHKGPGPGEYAMASSAESSPGEGPGPGKPKGLAYVEYENESGPGPGESVIQNYNKALQQLGPGPGHHHHHH**

**93**

**MAKLSTDELLDAFKEMTLLELSDFVKKFEETFEVTAAAPVAVAAAGAAPAGAAVEAAEEQSEFDVILEAAGDKKIGVIKVVREIVSGLGLKEAKDLVDGAPKPLLEKVAKEAADEAKAKLEAAGATVTVKAEAAAKEAAAKEAAAKAYPEHVCEVLAAYRNCPWTVALWAAYNPDFKVFRYAAYRLAEYQAYIAAYHVYDLFEKAAAYRLEKVHSLFRGPGPGVKDLRLVTNRAGKPKGPGPGKVFRYSTSLEKHKLGPGPGEYAMASSAESSPGEGPGPGFKVFRYSTSLEKHKGPGPGKPKGLAYVEYENESGPGPGESVIQNYNKALQQLGPGPGHHHHHH**

**94**

**MAKLSTDELLDAFKEMTLLELSDFVKKFEETFEVTAAAPVAVAAAGAAPAGAAVEAAEEQSEFDVILEAAGDKKIGVIKVVREIVSGLGLKEAKDLVDGAPKPLLEKVAKEAADEAKAKLEAAGATVTVKAEAAAKEAAAKEAAAKAYPEHVCEVLAAYRNCPWTVALWAAYNPDFKVFRYAAYRLAEYQAYIAAYHVYDLFEKAAAYRLEKVHSLFRGPGPGVKDLRLVTNRAGKPKGPGPGKVFRYSTSLEKHKLGPGPGEYAMASSAESSPGEGPGPGKPKGLAYVEYENESGPGPGFKVFRYSTSLEKHKGPGPGESVIQNYNKALQQLGPGPGHHHHHH**

**95**

**MAKLSTDELLDAFKEMTLLELSDFVKKFEETFEVTAAAPVAVAAAGAAPAGAAVEAAEEQSEFDVILEAAGDKKIGVIKVVREIVSGLGLKEAKDLVDGAPKPLLEKVAKEAADEAKAKLEAAGATVTVKAEAAAKEAAAKEAAAKAYPEHVCEVLAAYRNCPWTVALWAAYNPDFKVFRYAAYRLAEYQAYIAAYHVYDLFEKAAAYRLEKVHSLFRGPGPGVKDLRLVTNRAGKPKGPGPGKVFRYSTSLEKHKLGPGPGEYAMASSAESSPGEGPGPGKPKGLAYVEYENESGPGPGESVIQNYNKALQQLGPGPGFKVFRYSTSLEKHKGPGPGHHHHHH**

**96**

**MAKLSTDELLDAFKEMTLLELSDFVKKFEETFEVTAAAPVAVAAAGAAPAGAAVEAAEEQSEFDVILEAAGDKKIGVIKVVREIVSGLGLKEAKDLVDGAPKPLLEKVAKEAADEAKAKLEAAGATVTVKAEAAAKEAAAKEAAAKAYPEHVCEVLAAYRNCPWTVALWAAYNPDFKVFRYAAYRLAEYQAYIAAYHVYDLFEKAAAYRLEKVHSLFRGPGPGFKVFRYSTSLEKHKGPGPGVKDLRLVTNRAGKPKGPGPGKVFRYSTSLEKHKLGPGPGEYAMASSAESSPGEGPGPGKPKGLAYVEYENESGPGPGESVIQNYNKALQQLGPGPGHHHHHH**

**97**

**MAKLSTDELLDAFKEMTLLELSDFVKKFEETFEVTAAAPVAVAAAGAAPAGAAVEAAEEQSEFDVILEAAGDKKIGVIKVVREIVSGLGLKEAKDLVDGAPKPLLEKVAKEAADEAKAKLEAAGATVTVKAEAAAKEAAAKEAAAKAYPEHVCEVLAAYRNCPWTVALWAAYNPDFKVFRYAAYRLAEYQAYIAAYHVYDLFEKAAAYRLEKVHSLFRGPGPGFKVFRYSTSLEKHKGPGPGKVFRYSTSLEKHKLGPGPGVKDLRLVTNRAGKPKGPGPGEYAMASSAESSPGEGPGPGKPKGLAYVEYENESGPGPGESVIQNYNKALQQLGPGPGHHHHHH**

**98**

**MAKLSTDELLDAFKEMTLLELSDFVKKFEETFEVTAAAPVAVAAAGAAPAGAAVEAAEEQSEFDVILEAAGDKKIGVIKVVREIVSGLGLKEAKDLVDGAPKPLLEKVAKEAADEAKAKLEAAGATVTVKAEAAAKEAAAKEAAAKAYPEHVCEVLAAYRNCPWTVALWAAYNPDFKVFRYAAYRLAEYQAYIAAYHVYDLFEKAAAYRLEKVHSLFRGPGPGFKVFRYSTSLEKHKGPGPGKVFRYSTSLEKHKLGPGPGEYAMASSAESSPGEGPGPGVKDLRLVTNRAGKPKGPGPGKPKGLAYVEYENESGPGPGESVIQNYNKALQQLGPGPGHHHHHH**

**99**

**MAKLSTDELLDAFKEMTLLELSDFVKKFEETFEVTAAAPVAVAAAGAAPAGAAVEAAEEQSEFDVILEAAGDKKIGVIKVVREIVSGLGLKEAKDLVDGAPKPLLEKVAKEAADEAKAKLEAAGATVTVKAEAAAKEAAAKEAAAKAYPEHVCEVLAAYRNCPWTVALWAAYNPDFKVFRYAAYRLAEYQAYIAAYHVYDLFEKAAAYRLEKVHSLFRGPGPGFKVFRYSTSLEKHKGPGPGKVFRYSTSLEKHKLGPGPGEYAMASSAESSPGEGPGPGKPKGLAYVEYENESGPGPGVKDLRLVTNRAGKPKGPGPGESVIQNYNKALQQLGPGPGHHHHHH**

**100**

**MAKLSTDELLDAFKEMTLLELSDFVKKFEETFEVTAAAPVAVAAAGAAPAGAAVEAAEEQSEFDVILEAAGDKKIGVIKVVREIVSGLGLKEAKDLVDGAPKPLLEKVAKEAADEAKAKLEAAGATVTVKAEAAAKEAAAKEAAAKAYPEHVCEVLAAYRNCPWTVALWAAYNPDFKVFRYAAYRLAEYQAYIAAYHVYDLFEKAAAYRLEKVHSLFRGPGPGFKVFRYSTSLEKHKGPGPGKVFRYSTSLEKHKLGPGPGEYAMASSAESSPGEGPGPGKPKGLAYVEYENESGPGPGESVIQNYNKALQQLGPGPGVKDLRLVTNRAGKPKGPGPGHHHHHH**

101

**MAKLSTDELLDAFKEMTLLELSDFVKKFEETFEVTAAAPVAVAAAGAAPAGAAVEAAEEQSEFDVILEAAGDKKIGVIKVVREIVSGLGLKEAKDLVDGAPKPLLEKVAKEAADEAKAKLEAAGATVTVKAEAAAKEAAAKEAAAKARLEKVHSLFRAAYYPEHVCEVLAAYNPDFKVFRYAAYRLAEYQAYIAAYHVYDLFEKAAAYRNCPWTVALWGPGPGVKDLRLVTNRAGKPKGPGPGFKVFRYSTSLEKHKGPGPGKVFRYSTSLEKHKLGPGPGEYAMASSAESSPGEGPGPGKPKGLAYVEYENESGPGPGESVIQNYNKALQQLGPGPGHHHHHH**

**102**

**MAKLSTDELLDAFKEMTLLELSDFVKKFEETFEVTAAAPVAVAAAGAAPAGAAVEAAEEQSEFDVILEAAGDKKIGVIKVVREIVSGLGLKEAKDLVDGAPKPLLEKVAKEAADEAKAKLEAAGATVTVKAEAAAKEAAAKEAAAKARLEKVHSLFRAAYYPEHVCEVLAAYNPDFKVFRYAAYRLAEYQAYIAAYHVYDLFEKAAAYRNCPWTVALWGPGPGVKDLRLVTNRAGKPKGPGPGKVFRYSTSLEKHKLGPGPGFKVFRYSTSLEKHKGPGPGEYAMASSAESSPGEGPGPGKPKGLAYVEYENESGPGPGESVIQNYNKALQQLGPGPGHHHHHH**

**103**

**MAKLSTDELLDAFKEMTLLELSDFVKKFEETFEVTAAAPVAVAAAGAAPAGAAVEAAEEQSEFDVILEAAGDKKIGVIKVVREIVSGLGLKEAKDLVDGAPKPLLEKVAKEAADEAKAKLEAAGATVTVKAEAAAKEAAAKEAAAKARLEKVHSLFRAAYYPEHVCEVLAAYNPDFKVFRYAAYRLAEYQAYIAAYHVYDLFEKAAAYRNCPWTVALWGPGPGVKDLRLVTNRAGKPKGPGPGKVFRYSTSLEKHKLGPGPGEYAMASSAESSPGEGPGPGFKVFRYSTSLEKHKGPGPGKPKGLAYVEYENESGPGPGESVIQNYNKALQQLGPGPGHHHHHH**

**104**

**MAKLSTDELLDAFKEMTLLELSDFVKKFEETFEVTAAAPVAVAAAGAAPAGAAVEAAEEQSEFDVILEAAGDKKIGVIKVVREIVSGLGLKEAKDLVDGAPKPLLEKVAKEAADEAKAKLEAAGATVTVKAEAAAKEAAAKEAAAKARLEKVHSLFRAAYYPEHVCEVLAAYNPDFKVFRYAAYRLAEYQAYIAAYHVYDLFEKAAAYRNCPWTVALWGPGPGVKDLRLVTNRAGKPKGPGPGKVFRYSTSLEKHKLGPGPGEYAMASSAESSPGEGPGPGKPKGLAYVEYENESGPGPGFKVFRYSTSLEKHKGPGPGESVIQNYNKALQQLGPGPGHHHHHH**

**105**

**MAKLSTDELLDAFKEMTLLELSDFVKKFEETFEVTAAAPVAVAAAGAAPAGAAVEAAEEQSEFDVILEAAGDKKIGVIKVVREIVSGLGLKEAKDLVDGAPKPLLEKVAKEAADEAKAKLEAAGATVTVKAEAAAKEAAAKEAAAKARLEKVHSLFRAAYYPEHVCEVLAAYNPDFKVFRYAAYRLAEYQAYIAAYHVYDLFEKAAAYRNCPWTVALWGPGPGVKDLRLVTNRAGKPKGPGPGKVFRYSTSLEKHKLGPGPGEYAMASSAESSPGEGPGPGKPKGLAYVEYENESGPGPGESVIQNYNKALQQLGPGPGFKVFRYSTSLEKHKGPGPGHHHHHH**

**106**

**MAKLSTDELLDAFKEMTLLELSDFVKKFEETFEVTAAAPVAVAAAGAAPAGAAVEAAEEQSEFDVILEAAGDKKIGVIKVVREIVSGLGLKEAKDLVDGAPKPLLEKVAKEAADEAKAKLEAAGATVTVKAEAAAKEAAAKEAAAKARLEKVHSLFRAAYYPEHVCEVLAAYNPDFKVFRYAAYRLAEYQAYIAAYHVYDLFEKAAAYRNCPWTVALWGPGPGFKVFRYSTSLEKHKGPGPGVKDLRLVTNRAGKPKGPGPGKVFRYSTSLEKHKLGPGPGEYAMASSAESSPGEGPGPGKPKGLAYVEYENESGPGPGESVIQNYNKALQQLGPGPGHHHHHH**

**107**

**MAKLSTDELLDAFKEMTLLELSDFVKKFEETFEVTAAAPVAVAAAGAAPAGAAVEAAEEQSEFDVILEAAGDKKIGVIKVVREIVSGLGLKEAKDLVDGAPKPLLEKVAKEAADEAKAKLEAAGATVTVKAEAAAKEAAAKEAAAKARLEKVHSLFRAAYYPEHVCEVLAAYNPDFKVFRYAAYRLAEYQAYIAAYHVYDLFEKAAAYRNCPWTVALWGPGPGFKVFRYSTSLEKHKGPGPGKVFRYSTSLEKHKLGPGPGVKDLRLVTNRAGKPKGPGPGEYAMASSAESSPGEGPGPGKPKGLAYVEYENESGPGPGESVIQNYNKALQQLGPGPGHHHHHH**

**108**

**MAKLSTDELLDAFKEMTLLELSDFVKKFEETFEVTAAAPVAVAAAGAAPAGAAVEAAEEQSEFDVILEAAGDKKIGVIKVVREIVSGLGLKEAKDLVDGAPKPLLEKVAKEAADEAKAKLEAAGATVTVKAEAAAKEAAAKEAAAKARLEKVHSLFRAAYYPEHVCEVLAAYNPDFKVFRYAAYRLAEYQAYIAAYHVYDLFEKAAAYRNCPWTVALWGPGPGFKVFRYSTSLEKHKGPGPGKVFRYSTSLEKHKLGPGPGEYAMASSAESSPGEGPGPGVKDLRLVTNRAGKPKGPGPGKPKGLAYVEYENESGPGPGESVIQNYNKALQQLGPGPGHHHHHH**

**109**

**MAKLSTDELLDAFKEMTLLELSDFVKKFEETFEVTAAAPVAVAAAGAAPAGAAVEAAEEQSEFDVILEAAGDKKIGVIKVVREIVSGLGLKEAKDLVDGAPKPLLEKVAKEAADEAKAKLEAAGATVTVKAEAAAKEAAAKEAAAKARLEKVHSLFRAAYYPEHVCEVLAAYNPDFKVFRYAAYRLAEYQAYIAAYHVYDLFEKAAAYRNCPWTVALWGPGPGFKVFRYSTSLEKHKGPGPGKVFRYSTSLEKHKLGPGPGEYAMASSAESSPGEGPGPGKPKGLAYVEYENESGPGPGVKDLRLVTNRAGKPKGPGPGESVIQNYNKALQQLGPGPGHHHHHH**

**110**

**MAKLSTDELLDAFKEMTLLELSDFVKKFEETFEVTAAAPVAVAAAGAAPAGAAVEAAEEQSEFDVILEAAGDKKIGVIKVVREIVSGLGLKEAKDLVDGAPKPLLEKVAKEAADEAKAKLEAAGATVTVKAEAAAKEAAAKEAAAKARLEKVHSLFRAAYYPEHVCEVLAAYNPDFKVFRYAAYRLAEYQAYIAAYHVYDLFEKAAAYRNCPWTVALWGPGPGFKVFRYSTSLEKHKGPGPGKVFRYSTSLEKHKLGPGPGEYAMASSAESSPGEGPGPGKPKGLAYVEYENESGPGPGESVIQNYNKALQQLGPGPGVKDLRLVTNRAGKPKGPGPGHHHHHH**
